# Hippocampal long‐range GABAergic neurons target hippocampal‐projecting cells of the supramammillary area

**DOI:** 10.1113/JP291809

**Published:** 2026-09-11

**Authors:** Lauren Glassburn, Edmund Havener, Olivia Stangler, Ezequiel Marron Fernandez de Velasco, Esther Krook‐Magnuson

**Affiliations:** ^1^ Graduate Program in Neuroscience University of Minnesota Minneapolis Minnesota USA; ^2^ Department of Neuroscience University of Minnesota Minneapolis Minnesota USA; ^3^ Department of Pharmacology University of Minnesota Minneapolis Minnesota USA

**Keywords:** CA1, CA2, CA3, dentate, hypothalamus, inhibition, nNOS, septo‐temporal axis

## Abstract

**Abstract:**

The supramammillary area (SuM) is well known for its direct connections to, and varied impacts on, the hippocampus. In contrast, relatively little is known about connectivity in the reverse direction – that is, the hippocampal influence on the SuM. We find that the hippocampus provides direct GABAergic inputs to the SuM, arising from multiple hippocampal subregions across dorsal and ventral hippocampus. Although both nitric oxide synthase‐expressing long‐range inhibitory NOS^+^ cells (LINCs) of the hippocampus and somatostatin‐expressing subpopulations contribute to hippocampal‐SuM communication, hippocampal cells projecting to the SuM do not express markers common to other subsets of hippocampal inhibitory projection neurons, including muscarinic cholinergic receptor 2 (M2R) or vasoactive intestinal protein (VIP). Viral tracing and optogenetic experiments indicate that hippocampal inhibitory cells densely target the lateral SuM and preferentially innervate SuM neurons that project back to the hippocampus. Notably this is not simple feedback inhibition, as there is cross‐talk between circuits – dorsal hippocampal cells inhibit not only SuM cells projecting to dorsal hippocampus but also those projecting to the ventral hippocampus, and the same is true of ventral hippocampal cells. Collectively this work establishes that hippocampal‐SuM circuitry is bidirectional and, in doing so, uncovers an additional role for hippocampal long‐range GABAergic neurons in interregional communication.

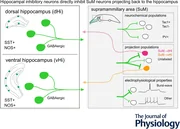

**Key points:**

- The supramammillary area (SuM) is a region of the hypothalamus with well‐established impacts on memory and oscillations via direct projections to the hippocampus. In comparison, little is known about hippocampal influence over the SuM.
- Hippocampal‐to‐SuM direct communication is mediated by hippocampal inhibitory neurons, including long‐range inhibitory NOS^+^ cells (LINCs) and somatostatin‐expressing cells.
- Hippocampal inhibitory neurons projecting to the SuM originate from across hippocampal subregions (hilus, CA3, CA2 and CA1) and from across the hippocampal dorsal–ventral axis.
- Hippocampal inhibitory neurons preferentially target SuM neurons which project back to the hippocampus, establishing that hippocampal‐SuM circuitry is bidirectional.

## Graphical abstract

**Abstract figure legend** The hippocampus contains a diversity of inhibitory neurons, including somatostatin‐expressing (SST^+^) and neuronal nitric oxide synthase‐expressing (NOS^+^) projection neurons that target extrahippocampal brain regions, including the hypothalamic supramammillary area (SuM). Hippocampal GABAergic projections to the SuM (Hi→SuM) originate from across the hippocampal subregions (including the hilus and CA3–CA1) and across the hippocampal dorsal–ventral axis (left panel). Although Hi→SuM neurons target a diversity of neurochemical SuM populations (right top), they preferentially target SuM neurons which project back to the hippocampus (right middle). Dorsal‐projecting (SuM→dHi) and ventral‐projecting (SuM→vHi) SuM populations are inhibited both by cells originating in the dorsal and by cells originating in the ventral hippocampus, allowing circuit cross‐communication. Hi→SuM neurons additionally preferentially target burst‐wave firing cells of the SuM (right bottom). Collectively this work expands our understanding of hippocampal inhibitory projection populations and establishes that the hippocampus directly influences SuM inputs to the hippocampus.

## Introduction

The supramammillary area (SuM) is a region of the hypothalamus with diverse functions (Billwiller et al., 2020; Escobedo et al., 2024; Farrell et al., 2021; Ito et al., 2018; Kesner et al., 2021; López‐Ferreras et al., 2020; Plaisier et al., 2020; Y. Li et al., 2022; Zhang et al., 2023). This includes known roles in modulating a range of hippocampal processes, including spatial and social memory (Chen et al., 2020; Qin et al., 2022; Robert et al., 2021; Y. Li et al., 2020). The SuM, and especially the lateral SuM, provides a number of direct connections to the hippocampus from neurochemically diverse SuM cells (Borhegyi & Leranth, 1997; Boulland et al., 2009; Jiang et al., 2024; Kesner et al., 2023; Luo et al., 2025; M. Li et al., 2023; Plaisier et al., 2020; Soussi et al., 2010; Tabuchi et al., 2022). For example, substance P‐expressing (Tac1^+^) SuM cells – which can project to the dentate gyrus (DG) and CA2 within the hippocampus – signal speed and can drive locomotion (Farrell et al., 2021). In contrast, SuM cells negative for Tac1 (Tac1^−^) and with collaterals to the medial septum fire phase‐locked to hippocampal oscillations and can control hippocampal spike timing within theta oscillations (Farrell et al., 2021). Projection‐defined SuM cells also show diverse functions. For example, SuM cells projecting to the dorsal hippocampus impact spatial memory, whereas SuM cells projecting to the ventral hippocampus impact affective behaviours (Luo et al., 2025).

Many of the brain regions receiving SuM input, including the medial septum, the interpeduncular nucleus and the periaqueductal grey, are reported to be reciprocally connected with the SuM (Chen et al., 2020; Farrell et al., 2024; Luo et al., 2025; Plaisier et al., 2020; Qin et al., 2022; Vertes, 1992a). However, although there has been increasing attention to the SuM's impact on the hippocampus, comparatively little attention has been given to the possible influence of the hippocampus on the SuM. This may be due to the lack of a traditional hippocampal connection to the SuM. Although areas including the subiculum do have excitatory projections to the SuM (Canteras & Swanson, 1992; Gonzalo‐Ruiz et al., 1999; Kishi et al., 2000; Tang et al., 2016), this does not appear to be the case for the hippocampus proper. Notably one study suggested that CA2 pyramidal neurons project to the SuM (Cui et al., 2013), but other research failed to find evidence of CA2 pyramidal projections to the SuM (Hitti & Siegelbaum, 2014). An early tracing study instead found non‐pyramidal neurons in the hippocampus labelled after horseradish peroxidase injection into the SuM (Ino, Itoh, Kamiya, et al., 1988), raising the possibility that the hippocampus does provide direct input to the SuM, but that this arises from inhibitory neurons.

Supporting inhibitory connections from the hippocampus to the SuM, previous work found that hippocampal LINCs – so called because they are long‐range inhibitory neuronal nitric oxide synthase positive (NOS^+^) cells – project to several extrahippocampal regions, including the SuM (Christenson Wick et al., 2019). More recently, work has noted that a population of medial septum‐projecting somatostatin (SST)‐expressing neurons in the dorsal dentate and CA3 regions have collaterals to the SuM (Kinney et al., 2025). In addition to these cell populations the hippocampus hosts a variety of inhibitory long‐range‐projecting cell types, typically characterized by their projection patterns and molecular markers, including parvalbumin (PV)‐expressing neurons with commissural projections to the contralateral hippocampus (Christenson Wick et al., 2017; Goodman & Sloviter, 1992; Zappone & Sloviter, 2001), vasoactive intestinal peptide (VIP)‐expressing neurons with projections to the subiculum (Francavilla et al., 2018), and a broader inhibitory population of muscarinic cholinergic receptor 2 (M2R)‐expressing TORO (theta‐off, ripple‐on) cells, which project to a range of extrahippocampal regions, including the medial septum and entorhinal cortex (Hájos et al., 1997; Miyashita & Rockland, 2007; Szabo et al., 2022). However whether VIP, PV, or M2R‐expressing cells can also project to the SuM is unknown.

We find that hippocampal inhibitory projections to the SuM (Hi→SuM cells) are found in the dentate hilus and across hippocampal CA regions, primarily in the stratum oriens, and are spread throughout the dorsal–ventral hippocampal axis. Hippocampal projections to the SuM arise from both NOS^+^ and SST^+^ cells, without a substantial contribution from PV, VIP, or M2R^+^ cells. We also did not find any evidence for excitatory projections to the SuM from the hippocampus proper. Inhibitory Hi→SuM projections densely innervate lateral (rather than medial) SuM, where they preferentially target hippocampally projecting SuM neurons. This positions Hi→SuM cells to regulate the SuM's influence on the hippocampus. The postsynaptic targets of Hi→SuM cells are neurochemically and electrophysiologically diverse but are enriched in cells displaying a burst‐wave firing pattern. Overall we find that Hi→SuM cells are positioned to directly inhibit SuM projections to the hippocampus, broadening our appreciation of hippocampal long‐range inhibitory neurons and adding a hitherto unstudied connection in hippocampal‐SuM interactions.

## Results

### Inhibitory neurons from across hippocampal subregions project to the SuM

**Figure 1:**
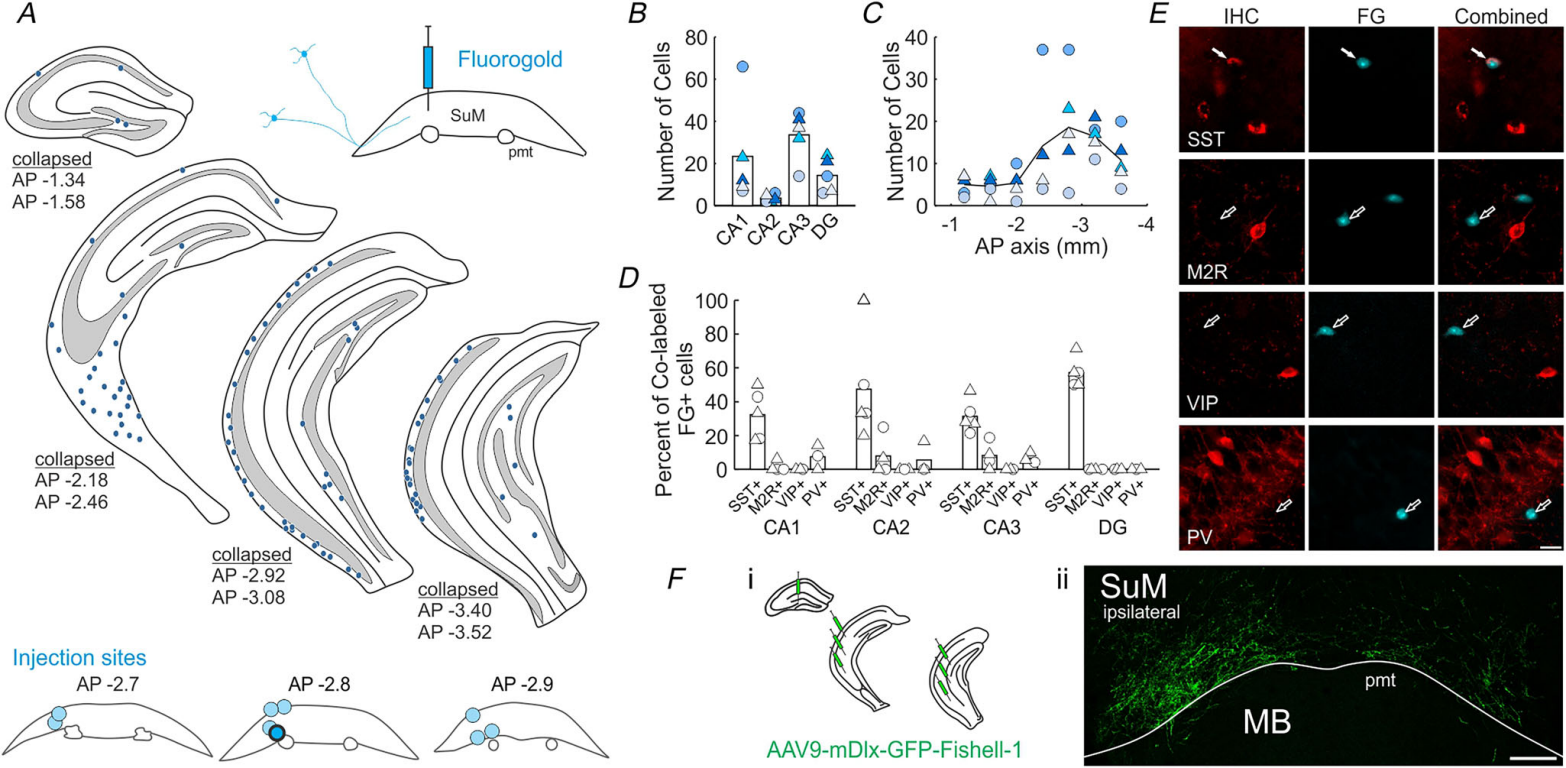
Cells from across hippocampal subregions project to the supramammillary area (SuM) *A*, fluorogold‐labelled (FG^+^) cells were identified across regions of the mouse hippocampus after injection of fluorogold into the SuM (schematic inset). Example cell locations are shown for one mouse, with two 50 µm‐thick sections from a 1‐in‐4 series of hippocampus collapsed per schematic. Injection sites are shown for each mouse (bottom), with the example mouse emphasized in dark blue. (*B*, *C*) FG^+^ cells were quantified by subregion (*B*) and by anterior–posterior (AP) location (*C*). *D*, percentage of FG^+^ cells immunopositive for SST, M2R, VIP, and PV by hippocampal region. *E*, FG^+^ cells were often SST immunopositive (e.g. double‐labelled cell in top row, filled arrow) but typically M2R, VIP, and PV immunonegative (open arrows). Scale bar: 20 µm. *F*, AAV‐mDlx‐GFP was injected throughout the hippocampus (schematic in (i)), allowing visualization of hippocampal inhibitory fibres in the SuM. Fibres were visible spanning both ipsilateral and contralateral SuM (ii), with stronger innervation present in the ipsilateral SuM and only very sparse innervation present in the medial SuM. Scale bar: 200 µm. Bars: mean across animals. Symbols: individual mouse; circles: females; triangles: males. IHC, immunohistochemistry; SuM, supramammillary; pmt, principal mammillary tract; MB, mammillary bodies.

The hippocampus is a highly organized structure, with circuits and functions varying along multiple axes (Cembrowski et al., 2016; Fanselow & Dong, 2010; Kheirbek et al., 2013; Krook‐Magnuson et al., 2012; Okamoto et al., 2025; Strange et al., 2014; Valero & de la Prida, 2018; Van Strien et al., 2009). To identify which regions and layers Hi→SuM neurons are located we injected a small volume (10 nL) of the retrograde tracer Fluorogold (FG) into the SuM and examined FG^+^ cells across the septo‐temporal (anterior–posterior (AP)) axis of the hippocampus (Fig. 1). Hi→SuM neurons were not restricted to one area within the hippocampus. Instead we identified FG^+^ neurons across CA1, CA2, CA3 and the DG hilus (*n* = 5 mice) (Fig. 1*A*,*B*). Hi→SuM neurons were especially numerous in the stratum oriens (Fig. 1*A*). The location of FG^+^ cells is therefore consistent with previous literature suggesting that Hi→SuM projections arise from non‐pyramidal cells (Ino, Itoh, Kamiya, et al., 1988). FG^+^ cells were distributed across the AP axis of the hippocampus, with increasing numbers found in more posterior sections (Fig. 1*A*,*C*).

Long‐range‐projecting inhibitory cells of the hippocampus have been characterized by their expression of a diverse set of markers (Jinno et al., 2007; Katona et al., 2017; Melzer et al., 2012), including SST (characteristic of hippocampal‐septal cells) (Booker & Vida, 2018; Jinno, 2009; Jinno & Kosaka, 2002; Jinno et al., 2007; Kinney et al., 2025; Yuan et al., 2017), PV (expressed by some commissurally projecting cells) (Christenson Wick et al., 2017; Goodman & Sloviter, 1992; Zappone & Sloviter, 2001), VIP (expressed by some hippocampal‐subiculum inhibitory projections) (Francavilla et al., 2018), and M2R (expressed by extrahippocampal‐projecting TORO cells) (Hájos et al., 1997; Miyashita & Rockland, 2007; Szabo et al., 2022). To examine which hippocampal populations beyond LINCs may project to the SuM we examined the immunohistochemistry profile of retrogradely labelled hippocampal cells. Across all hippocampal subregions, a substantial percentage of FG^+^ Hi→SuM cells were SST immunopositive (CA1: 32.4 ± 6.5% [SD: 14.6%]; CA2: 47.3 ± 14% [SD: 31.3%]; CA3: 31.4 ± 4.2% [SD: 9.5%]; DG: 57.1 ± 3.9% [SD: 8.7%]; *n* = 5 mice; Fig. 1*D*,*E*). In contrast, almost no FG^+^ Hi→SuM cells were immunopositive for M2R (CA1: 1.5 ± 1.5% [SD: 3.0%]; CA2: 7.9 ± 5.9% [SD: 11.8%]; CA3: 8.2 ± 4% [SD: 7.9%]; DG: 0 ± 0.0% [SD: 0.0%]; *n* = 4 mice; Fig. 1*D*,*E*) or PV (CA1: 7.4 ± 4.1% [SD: 7.2%]; CA2: 5.6 ± 5.6% [SD: 9.6%]; CA3: 6.6 ± 1.8% [SD: 3.0%]; DG: 0.0 ± 0.0% [SD: 0.0%]; *n* = 3 mice; Fig. 1*D*,*E*), and no FG^+^ Hi→SuM cells were immunopositive for VIP (0.0 ± 0.0% [SD: 0.0%] for all subregions; *n* = 3 mice; Fig. 1*D*,*E*). Thus a large percentage of Hi→SuM cells are SST‐immunopositive cells but are not M2R^+^ TORO, VIP^+^, nor PV^+^ cells.

The mDlx enhancer can be used to specifically target inhibitory neurons in the forebrain, including the hippocampus (Dimidschstein et al., 2016). To see anterograde hippocampal inhibitory projections in the SuM we injected AAV9‐mDlx‐GFP at multiple sites across the hippocampus (Fig. 1*Fi*). With this broad capture of hippocampal inhibitory neurons, we observed fibres densely innervating the ipsilateral SuM, more sparsely innervating the contralateral SuM, and showing very sparse innervation of the medial SuM (Fig. 1*Fii*). This further confirms that inhibitory neurons of the hippocampus project to the SuM and suggests strong ipsilateral innervation of the SuM subregion (i.e. lateral SuM) which sends dense projections to the hippocampus (Haglund et al., 1984; Ino, Itoh, Sugimoto, et al., 1988; Kiss et al., 2000; Pan & McNaughton, 2004; Qin et al., 2022; Segal & Landis, 1974; Soussi et al., 2010; Vertes, 1992b).

### Dorsal and ventral hippocampal SST^+^ and NOS^+^ cells project to the SuM

**Figure 2:**
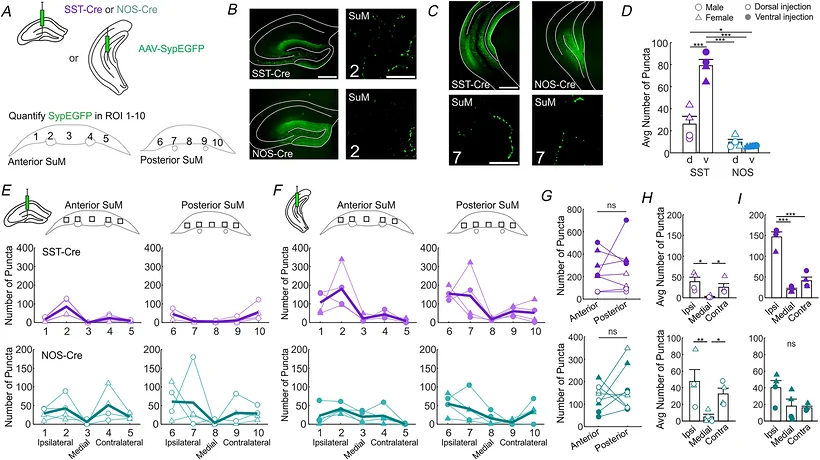
Hippocampal SST^+^ and NOS^+^ cells project to the supramammillary area (SuM) *A*, hippocampal virus injection schemes (top) and SuM regions of interest (ROIs) used for terminal quantification (bottom), numbered for reference. *B*, examples of viral expression at the injection site (left) and resulting labelled terminals within the SuM (right) in SST‐Cre (top) and NOS‐Cre (bottom) mice injected in the dorsal hippocampus. *C*, same as (*B*) but with the ventral hippocampus injected (left: SST‐Cre; right: NOS‐Cre). The ROI for each SuM example image is labelled in the bottom left corner. *D*, average number of puncta per ROI plotted by mouse line and injection scheme (*n* = 4 mice/scheme). Ventral hippocampal SST^+^ cells show the densest innervation of the SuM relative to all other schemes. All data in (*D*) were quantified with an automated pipeline. d, dorsal; v, ventral. *E*, number of terminals quantified in each SuM ROI in SST‐Cre (top, purple) and NOS‐Cre (bottom, teal) mice with injections in dorsal hippocampus (open symbols). Each point represents a single ROI from a single mouse. NOS‐Cre data in (*E*–*I*) were manually quantified. *F*, same as in *E* but for mice with injections in ventral hippocampus (closed symbols). *G*, total number of puncta across all five ROIs in the anterior or posterior SuM. Anterior and posterior SuM have similar levels of innervation from both hippocampal SST^+^ cells (top) and hippocampal NOS^+^ cells (bottom). *H*, *I*, average number of puncta in ipsilateral (ipsi; ROIs 1, 2, 6, 7), medial (ROIs 3, 8) and contralateral (contra; ROIs 4, 5, 9, 10) SuM. Both SST^+^ and NOS^+^ dorsal hippocampal cells (*H*) showed sparser innervation of medial SuM relative to both ipsilateral and contralateral SuM; ipsilateral and contralateral SuM have similar innervation from dorsal hippocampal cells; ventral hippocampal SST^+^ cells (top, I) preferentially innervate ipsilateral SuM compared to medial or contralateral SuM. Bars and bold lines: average across animals. Error bars: SEM. Symbols: individual animals; circle: male; triangle: female. All *post hoc* comparisons were performed using Tukey‐Kramer tests (see Table A2). *P < 0.05; **P < 0.01; ***P < 0.001. ns, not significant. Scale bars: 500 µm (*B*, left; *C*, top); 20 µm (*B*, right; *C*, bottom).

**Figure A1:**
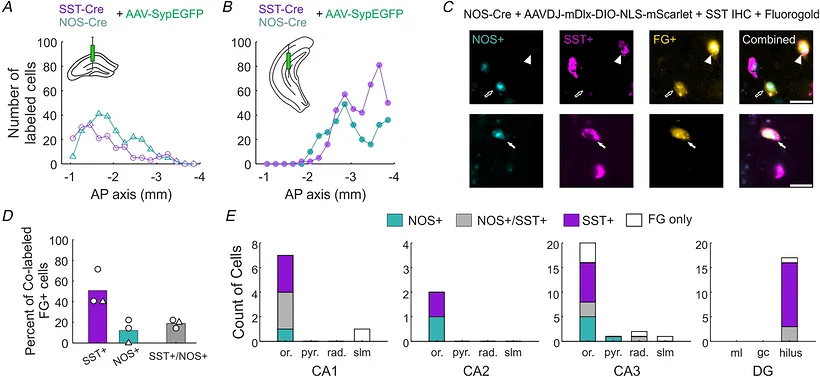
SST^+^ and NOS^+^ Hi→SuM cells are distributed throughout the hippocampus *A*, *B*, for imaged SST‐Cre and NOS‐Cre tissue in Fig. 2 *B*,*C* the number of virally labelled hippocampal cells across the anterior–posterior (AP) axis is quantified in dorsal‐injected (*A*) and ventral‐injected (*B*) SST‐Cre and NOS‐Cre mice (*n* = 1 mouse per scheme). Each point represents the number of labeled cells in a 50 µm thick section of hippocampus. *C*, in NOS‐Cre mice Hi→SuM neurons were retrogradely labelled via fluorogold (FG) injection into the SuM, and NOS^+^ cells were labelled via Cre‐dependent AAV (Adeno‐associated virus). SST immunohistochemistry (IHC) was then applied, yielding tissue with retrogradely labelled Hi→SuM neurons (yellow), fluorescent NOS^+^‐labelled cells at virus injection sites (teal) and labelled SST immunopositive cells (magenta). Example images show a solely SST^+^/FG^+^ cell (arrowhead, top), a solely NOS^+^/FG^+^ cell (open arrow, top) and a dual‐labelled SST^+^/NOS^+^/FG^+^ cell (closed arrow, bottom). Scale bar: 20 µm. *D*, percentage of FG^+^ cells which were SST^+^ (purple), NOS^+^ (teal) or dual NOS^+^/SST^+^ (grey) were counted. Quantified cells were restricted to regions with adequate viral spread for identifying NOS^+^ neurons (*n* = 3 mice). *E*, Hi→SuM cell types were counted and categorized by hippocampal subregion and sublayer, pooling together data from all three mice. Overall the largest category of cells was solely SST immunopositive (25/51, 49.0% of all cells counted) and fewer were SST immunopositive and NOS^+^ (10/51, 19.6% of all cells) or SST immunonegative and NOS^+^ (8/51, 15.7% of all cells). There were additionally solely FG^+^ cells that did not show SST or NOS expression (white) (8/51, 15.7% of all cells counted). circle – female; triangle – male or stratum oriens; pyr – stratum pyramidale; rad – stratum radiatum; slm – stratum lacunosum moleculare; ml – molecular layer; gc – granule cell layer.

The FG work described above indicates that a large percentage of Hi→SuM cells express SST. Previous work has shown that there are also LINCs in the hippocampus projecting to the SuM (Christenson Wick et al., 2019). LINCs can express NOS in dendrites without observed somatic immunolabelling (Burette et al., 2002; Christenson Wick et al., 2019); as FG labelling is observed largely in the soma, with poor labelling in neurites, quantifying the contribution of LINCs to Hi→SuM projections is not possible using a traditional approach based on immunolabeling of FG^+^ cells. Therefore to compare hippocampal NOS^+^ and SST^+^ cells’ innervation of the SuM, we used a Cre‐dependent AAV to express EGFP‐tagged synaptophysin (SypEGFP) in NOS‐Cre or SST‐Cre mice. Tagging to synaptophysin supports trafficking of EGFP to the presynaptic terminal, allowing the visualization and quantification of innervation of the SuM (Oh et al., 2014). We additionally aimed to compare innervation of the SuM by the dorsal hippocampus (dHi) and the ventral hippocampus (vHi), focusing on input primarily from the dentate in each region, as the dentate gyrus is a strong recipient of SuM input (Boulland et al., 2009; Haglund et al., 1984; Soussi et al., 2010; Vertes, 1992b). Therefore 3 weeks after viral injection into the dHi or vHi, we harvested the tissue and quantified SypEGFP+ puncta across the SuM, including anterior, posterior, medial and lateral SuM, with one of four subsets of hippocampal cells expressing SypEGFP: dHi SST^+^ cells, vHi SST^+^ cells, dHi NOS^+^ cells, or vHi NOS^+^ cells (*n* = 4 mice/scheme) (Fig. 2; Fig. A1).

Paralleling the immunohistochemistry work there was heavy innervation of the SuM from hippocampal SST^+^ cells (Fig. 2*B* top, 2*C* left). Matching previous work, which found innervation of the SuM by LINCs, there was also notable innervation of the SuM by NOS^+^ cells (Fig. 2*B* bottom, 2*C* right). Overall there was significantly greater innervation of the SuM by hippocampal SST^+^ neurons, reflected as a greater number of SypEGFP+ puncta (repeated measures, mixed‐effects ANOVA; NOS^+^ *vs*. SST^+^ cells, main effect, *F*_(1,10)_ = 76.58, *P* < 0.001; Table A1). SuM innervation from SST^+^ cells was especially strong from the ventral hippocampus (dHi *vs*. vHi, main effect, *F*_(1,10)_ = 23.13, *P* < 0.001; significant interaction between hippocampal cell type and hippocampal region, *F*_(1,10)_ = 30.89, *P* < 0.001; Table A1), such that SST^+^ vHi inhibitory projections showed the densest innervation of the SuM relative to all other schemes (*P* < 0.001 all comparisons, *post hoc* Tukey‐Kramer (TK); Table A2; Fig. 2*D*). There was no significant effect of sex on SuM innervation (main effect, *F*_(1,10)_ = 0.24, *P* = 0.637; Table A1).

The SuM itself has subdivisions and topographical organization. For example the medial SuM contains populations of dopaminergic cells (Gonzalo‐Ruiz et al., 1992; Plaisier et al., 2020; Swanson, 1982) and solely GABAergic cells (Allen Institute & Zeng, 2023; Soussi et al., 2010; Yao et al., 2023), and has been associated with functions including appetite regulation (Le May et al., 2019; Plaisier et al., 2020; Vogel et al., 2016; Zhang et al., 2023); in contrast the majority of hippocampally projecting SuM cells are located more laterally in areas near the principal mammillary tract (Haglund et al., 1984; Ino, Itoh, Sugimoto, et al., 1988; Pan & McNaughton, 2004; Segal & Landis, 1974; Vertes, 1992b). We therefore analysed hippocampal innervation of specific regions of interest (ROIs) in the SuM, namely anterior *vs*. posterior SuM and lateral *vs*. medial SuM, including comparing Hi→SuM ipsilateral and contralateral projection terminals.

Overall we found no significant difference in hippocampal innervation of anterior *vs*. posterior SuM (main effect, *F*_(1,10)_ = 0.28, *P* = 0.607; Table A1; SST anterior *vs*. posterior, *P* = 0.680, TK; NOS anterior *vs*. posterior, *P* = 0.571, TK; Table A2; Fig. 2*G*). In contrast we did find significant differences in hippocampal innervation across medial–lateral subregions of the SuM (main effect *F*_(2,20)_ = 92.32, *P* < 0.001; Table A1; Fig. 2*H*,*I*). Both dHi SST^+^ and dHi NOS^+^ cells showed sparser innervation of medial SuM relative to both ipsilateral and contralateral SuM (dHi SST^+^: ipsilateral *vs*. medial SuM, *P* = 0.0149; contralateral *vs*. medial SuM, *P* = 0.0438, TK; dHi NOS^+^: ipsilateral *vs*. medial, *P* = 0.00543; contralateral *vs*. medial, *P* = 0.0164, TK; Table A2; Fig. 2*E*,*H*). Interestingly dorsal hippocampal projections, regardless of the cell type, showed similar innervation of ipsi‐ and contralateral SuM (SST^+^ dHi: ipsilateral *vs*. contralateral, *P* = 0.574, TK; NOS^+^ dHi: ipsilateral *vs*. contralateral, *P* = 0.505, TK; Table A2; Fig 2*E*,*H*). In contrast vHi SST^+^ cells showed strong preferential innervation of ipsilateral SuM (ipsilateral *vs*. medial, *P* < 0.001, TK; ipsilateral *vs*. contralateral, *P* < 0.001, TK; Table A2; Fig. 2*F*,*I*, top), suggesting that the stronger overall hippocampal innervation of the ipsilateral SuM (Fig. 1*F*) is heavily mediated by SST^+^ vHi hippocampal neurons.

Collectively these data indicate broad hippocampal innervation of the SuM, with the densest innervation arising from SST^+^ cells from ventral hippocampus targeting the lateral SuM.

Although the majority of NOS^+^ LINCs do not express SST, a small subset does (<10% of LINCs coexpress SST) (Christenson Wick et al., 2019). To assess the extent to which NOS^+^ and SST^+^ Hi→SuM cells may be overlapping populations, we virally labelled hippocampal NOS^+^ cells and overlaid this with SST immunohistochemistry and FG retrograde tracing. Specifically NOS‐Cre mice were injected with an AAVDJ‐mDlx‐DIO‐NLS‐mScarlet vector to label nuclei of inhibitory NOS^+^ cells across the hippocampus (*n* = 3 mice). Virus was allowed to incubate for ∼2 weeks before FG was injected into the SuM for retrograde labelling of Hi→SuM cells. One week later we harvested tissue and performed SST immunohistochemistry. When quantifying virally labelled NOS^+^ cells and immunopositive SST^+^ cells, we found that retrogradely labelled Hi→SuM cells included both singly labelled SST^+^ cells and singly labelled NOS^+^ cells, suggesting that NOS^+^ Hi→SuM and SST^+^ Hi→SuM cells can be distinct cell populations (Fig. A1, panel *C*). Although there were some apparent regional differences, approximately half of retrogradely labelled cells were only SST^+^ overall, whereas approximately 16% were NOS^+^ but not SST^+^ (Fig. A1, panel *D*,*E*). Together with the terminal labelling results described above, this suggests overall greater innervation of the SuM by SST^+^ neurons. Importantly dual‐labelled SST^+^/NOS^+^ cells were also noted, with approximately one‐fifth of examined FG‐labelled Hi→SuM cells coexpressing SST and NOS (Fig. A1, panels *C–E*). Therefore this work suggests that there are neurochemically at least three populations of Hi→SuM cells: SST‐positive but NOS‐negative, NOS‐positive but SST‐negative, and SST and NOS coexpressing cells.

### Hippocampal inhibitory neurons target neurochemically diverse SuM cells, including Tac1^+^, Tac1^−^, and PV^+^ cells

**Figure 3:**
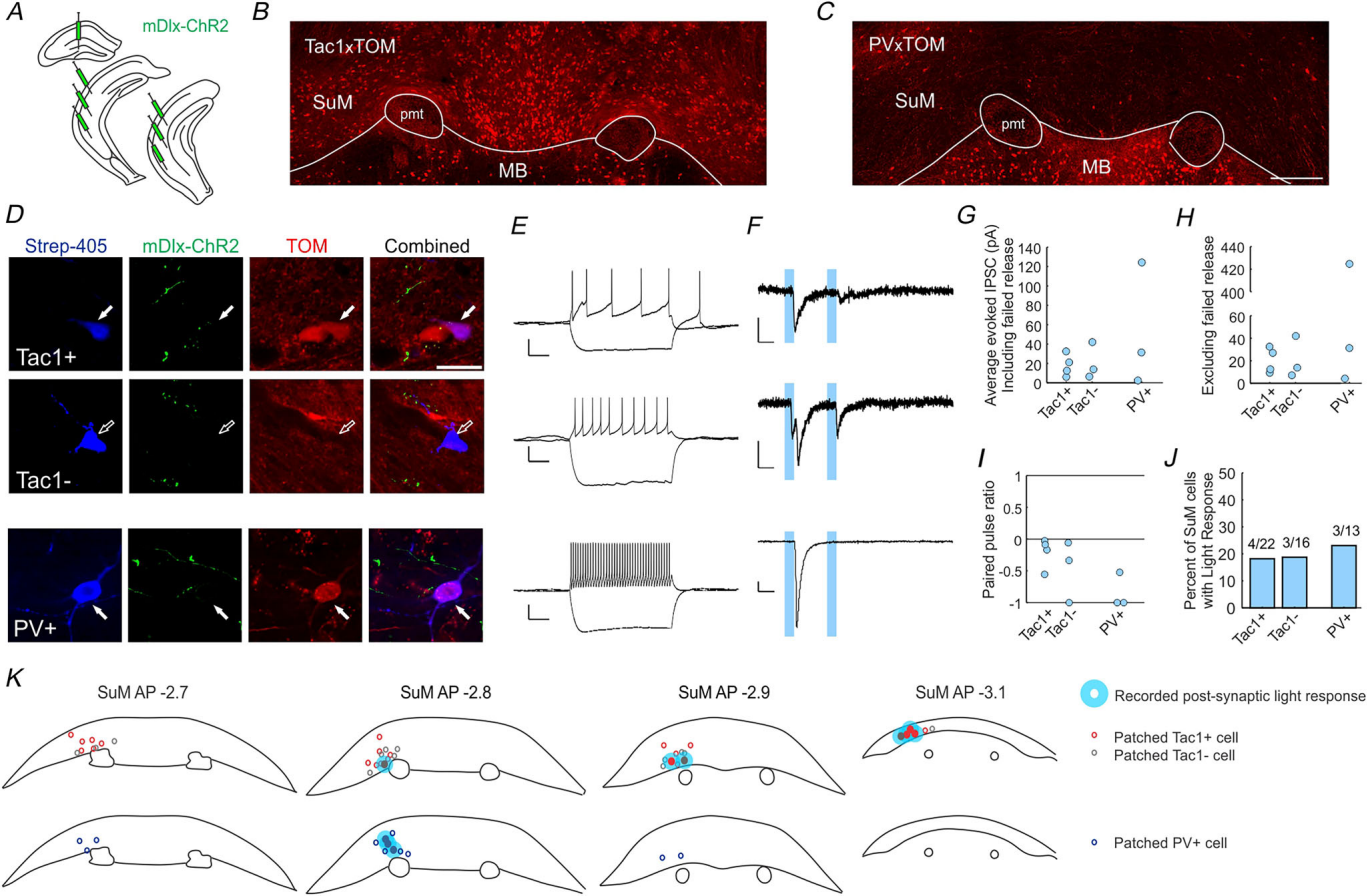
Hippocampal inhibitory neurons target neurochemically diverse supramammillary area (SuM) cells *A*, ChR2 was expressed in inhibitory neurons across the hippocampus in Tac1xTOM mice or in PV‐cre mice. *B*, *C*, images of TOM expression in either Tac1xTOM or PVxTOM transgenic mice. In Tac1xTOM mice fluorescence is found in many cells across the SuM (*B*), whereas only sparse fluorescent cells are present in the SuM in PVxTOM mice (*C*). Scale bars: 200 µm. *D*, Tac1^+^, Tac1^−^, and PV^+^ cells were patched, filled with biocytin and recovered with streptavidin‐405 (blue). Filled arrows point to a Tac1^+^ cell with recorded light‐evoked inhibitory postsynaptic currents (IPSCs) (top) and to a PV^+^ cell with recorded light‐evoked IPSCs (bottom). Outlined arrows point to a Tac1^−^ cell also displaying light‐evoked IPSCs (middle). Scale bar: 20 µm. *E*, cell firing was recorded while injecting a series of current steps. Example traces from cells corresponding to (*D*) are shown, for a −100 pA and a +100 pA step (top and bottom) or to a −100 pA and +25 pA step (middle). Scale bars: 20 mV, 100 ms. *F*, blue light was delivered to activate ChR2^+^ hippocampal inhibitory fibres. Example average traces of the recorded light‐evoked IPSCs are shown, corresponding to cells in (*D*). Scale bar: 20 pA, 10 ms, top and middle; 50 pA, 10 ms, bottom. *G*, *H*, the average amplitude of postsynaptic currents recorded in Tac1^+^, Tac1^−^, and PV^+^ cells; points represent the absolute value of the average evoked IPSCs for each recorded cell with a response, either including failed release events (*G*) or only including successful release events (*H*). *I*, the paired pulse ratio of responses to repeated blue light delivery, delivered at a 25 ms interval. All responses recorded in Tac1^+^, Tac1^−^, and PV^+^ cells showed paired pulse depression. *J*, the percentage of patched cells with a light‐evoked response. A comparable proportion of Tac1^+^_,_ Tac1^−^, and PV^+^ cells receive direct inhibition from the hippocampus. *K*, a map of patched Tac1^+^ (red, top), Tac1^−^ (grey, top), and PV^+^ (blue, bottom) cells. Cells were largely sampled from lateral SuM, where Hi→SuM fibres are concentrated. Cells with recorded light‐evoked responses are highlighted in blue. SuM, supramammillary; MB, mammillary bodies; pmt, principal mammillary tract.

**Figure A2:**
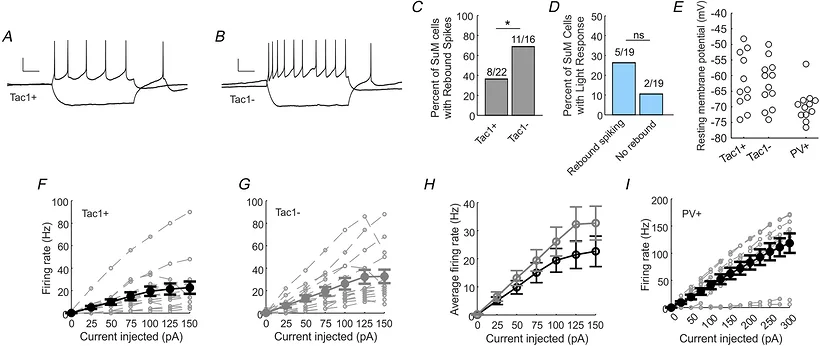
Firing properties of Tac1^+^, Tac1^−^, and PV^+^ cells *A*, *B*, cell firing was recorded while injecting a series of current steps. Example traces from a Tac1^+^ (*A*) and a Tac1^−^ (*B*) cell with rebound spiking are shown, for a −100 pA and a +100 pA step. Scale bar: 20 mV, 100 ms. *C*, the percentage of Tac1^+^ and Tac1^−^ cells with rebound spiking. More Tac1^−^ cells exhibited rebound spiking than Tac1^+^ cells (8/22, 36.4% of Tac1^+^ cells *vs*. 11/16, 68.8% of Tac1^−^ cells, χ^2^ = 3.89, *P* = 0.0487). *D*, proportion of cells with light responses, with pooled Tac1^+^ and Tac1^−^ populations separated into rebound spiking *vs*. non‐rebound spiking cells. There was no significant difference in innervation of these cell populations (*P* = 0.405, Fisher's exact test). *E*, resting membrane potential (RMP) recorded at break‐in in Tac1^+^, Tac1^−^ and PV^+^ neurons. When comparing Tac1^+^ and Tac1^−^ neurons, there was no significant difference between populations (−61.8 ± 2.7 mV, *n* = 11 Tac1^+^ cells, *vs*. −62.6 ± 2.2 mV, *n* = 12 Tac1^−^ cells, *P* = 0.95, Mann–Whitney *U* test). Cells that fired action potentials at rest were excluded from this analysis. *F*, *G*, the evoked firing rate for Tac1^+^ (*F*; *n* = 15 cells) and Tac1^−^ (*G*; *n* = 14 cells) cells when injecting current steps. Baseline current was injected to maintain the starting membrane voltage at −60 mV. *H*, the average evoked firing rate in Tac1^+^ (black) and Tac1^−^ (grey) neurons. There was no significant difference between the populations (repeated‐measures ANOVA, *F*_(1,27)_ = 1.23, *P* = 0.28). *I*, same as *F* and *G*, but for PV^+^ cells (*n* = 10 cells). Data correspond to experiments shown in Fig. 3. *P < 0.05. ns, not significant.

To determine if hippocampal GABAergic projections to the SuM make functional connections we performed whole‐cell patch‐clamp recordings from SuM neurons, with the excitatory opsin channelrhodopsin (ChR2) expressed broadly in GABAergic hippocampal neurons (Fig. 3). To further explore if specific SuM cell types are preferentially targeted by hippocampal inhibition we examined neurochemically defined subpopulations of SuM cells, focusing first on Tac1^+^ *vs*. Tac1^−^ cells. Both Tac1^+^ and Tac1^−^ cells can project to the hippocampus, among other areas, but Tac1^+^ cell activity and stimulation are closely tied to locomotion, whereas Tac1^−^ cells (and specifically those with collaterals to the medial septum) are instead closely tied to hippocampal theta and spike timing (Farrell et al., 2021). To examine if the hippocampus provides direct input to Tac1^+^ and/or Tac1^−^ cells we crossed Tac1‐Cre mice with a tdTomato (TOM) reporter line (Fig. 3*B*) and patched from both TOM‐expressing (i.e. Tac1^+^) and TOM‐negative (i.e. Tac1^−^) SuM neurons (Fig. 3*D*–*F*). Tac1^+^ and Tac1^−^ cells had largely similar *ex vivo* firing properties in our recordings (Figs 3*E* and A2), although there was a difference in the proportion of cells with rebound firing that just approached significance, with Tac1^−^ cells more commonly displaying rebound firing (8/22, 36.4% of Tac1^+^ cells *vs*. 11/16, 68.8% of Tac1^−^ cells with rebound firing, χ^2^ = 3.89, *P* = 0.0487, from *n* = 12 sections in 7 mice) (Fig. A2, panels *A–C*).

After the firing properties of the patched cell were recorded, we delivered brief pulses of blue light to the slice, allowing optogenetic activation of hippocampal inhibitory fibres in the SuM. As evidenced by light‐evoked postsynaptic responses in the patched cells, both Tac1^+^ and Tac1^−^ cells receive direct inhibition from the hippocampus (Fig. 3*F*). There was no preferential innervation of one population over the other, with a comparable proportion of patched Tac1^+^ and Tac1^−^ cells showing light‐evoked inhibitory postsynaptic currents (IPSCs) (light‐evoked IPSCs were observed in 4/22, 18.2% of Tac1^+^ cells *vs*. 3/16, 18.8% of Tac1^−^ cells, *P* = 1, Fisher's exact test). Light‐evoked IPSC amplitudes were also similar between patched Tac1^+^ and Tac1^−^ cells in lateral SuM, whether looking at all trials (absolute value IPSC amplitudes including failed release, Tac1^+^ cells: 18.0 ± 5.7 pA [SD: 11.5 pA]; Tac1^−^ cells: 20.8 ± 10.8 pA [SD: 18.8 pA]; *P* = 0.857, Mann–Whitney *U* test (MWU)) or examining only trials where light successfully evoked an IPSC (absolute value IPSC amplitudes excluding failed release, Tac1^+^ cells: 20.3 ± 5.6 pA [SD: 11.2 pA]; Tac1^−^ cells: 21.0 ± 10.7 pA [SD: 18.5 pA]; *P* = 1, MWU). To further examine synaptic properties, two light pulses 20 ms apart were delivered. Facilitating synapses will show an increased IPSC amplitude in response to the second pulse of light; depressing synapses will show a decreased IPSC amplitude in response to the second pulse of light. Light‐evoked responses in both Tac1^+^ or Tac1^−^ cells showed paired pulse depression, indicating a high initial probability of release at synapses onto either cell population (Fig. 3*I*).

Hippocampal inhibition may target fast‐spiking neurons of the SuM (Kinney et al., 2025), suggesting the possibility of the hippocampus directly suppressing SuM PV^+^ neurons. Although PV^+^ neurons have been reported absent from the SuM (L. Li et al., 2025), we observed sparse but present PV^+^ cells in the SuM of PVxTOM mice (Fig. 3*C*). To determine if SuM PV^+^ cells are indeed a fast‐spiking population and if they are targeted by hippocampal inhibition, we expressed AAV‐FLEX‐tdTomato in the SuM of PV‐Cre mice and expressed ChR2 in inhibitory cells across the hippocampus. We found that the majority of SuM PV^+^ cells are indeed fast‐spiking (maximum firing: 200 ± 25 Hz [SD: 90 Hz], *n* = 13 cells from four sections in two mice) (Fig. 3*E*, bottom; Fig. A2, panel *I*). Patched SuM PV^+^ cells received direct hippocampal inhibition (Fig. 3*D*–*I*) and at a similar proportion to patched Tac1^+^ and Tac1^−^ populations (3/13, 23.1% of PV^+^ cells showed light‐evoked IPSCs) (Fig. 3*J*).

These experiments demonstrate that Hi→SuM postsynaptic targets have diverse neurochemical identities, with approximately 20% of the patched Tac1^+^, Tac1^−^, and PV^+^ cells receiving hippocampal inhibitory input. These cells were sampled from lateral SuM (Fig. 3*K*), where Hi→SuM fibres are most dense (Figs 1, 2). Given that lateral SuM is where hippocampal‐projecting neurons are concentrated, we next sought to examine hippocampal inhibition of SuM populations defined by their projection targets, specifically those neurons with projections to the hippocampus.

### Hippocampal inhibitory neurons preferentially target SuM neurons which project back to the hippocampus

**Figure A3:**
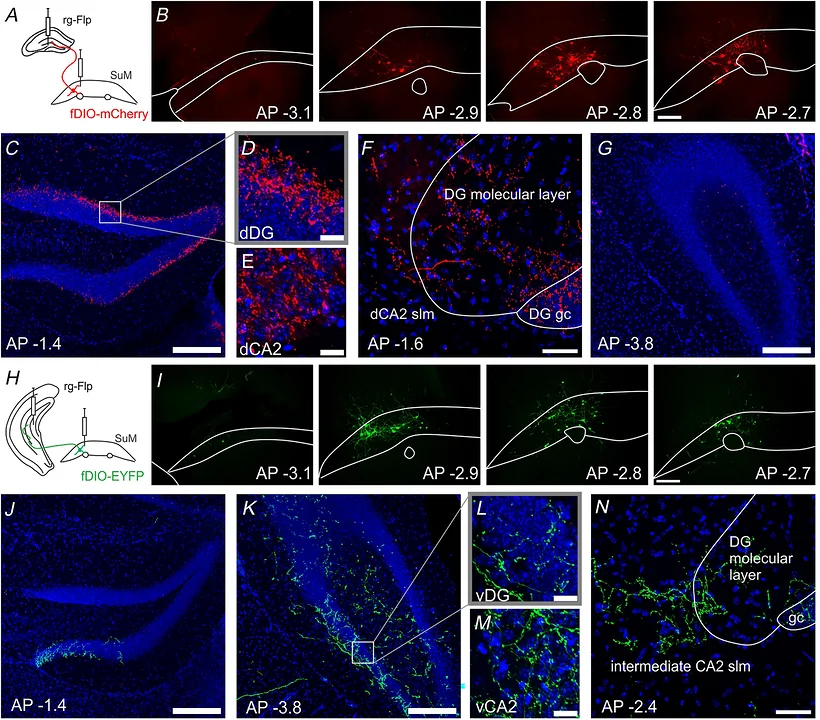
SuM→dHi and SuM→vHi neurons are largely separate populations *A*, retrograde AAV‐Flp was injected into dorsal hippocampus and a Flp‐dependent mCherry expressed in the SuM to label SuM neurons projecting to dorsal hippocampus (SuM→dHi). Injection co‐ordinates were the same as used for experiments using retrograde AAV‐mCherry in Fig. 4. *B*, series of SuM sections with SuM→dHi neurons labelled. Scale bar: 200 µm. *C*–*E*, SuM→dHi neurons project to dorsal dentate gyrus (dDG) (*C*, scale bar: 200 µm) and dorsal CA2. High magnification images of the fibres are shown for dDG (*D*) and dCA2 (*E*) (scale bar: 20 µm). *F*, some SuM→dHi projection fibres travel between the DG molecular layer and the CA2 stratum lacunosum moleculare (slm). Scale bar: 50 µm. *G*, SuM→dHi neurons do not densely innervate the ventral dentate gyrus. Scale bar: 200 µm. *H*, *I*, same as *A* and *B*, but a Flp‐dependent EYFP is expressed in SuM neurons projecting to ventral hippocampus (SuM→vHi). *J*, SuM→vHi neurons do not densely innervate the dorsal dentate gyrus. *K*, *L*, same as *C*–*E*, but showing SuM→vHi innervation of vDG and vCA2. *N*, same as (*F*), but looking at fibres crossing between intermediate DG molecular layer and CA2 slm. AP, anterior–posterior; gc, granule cell layer; slm, stratum lacunosum moleculare.

The function and connectivity of the hippocampus varies along the dorsal–ventral axis (Fanselow & Dong, 2010; Kishi et al., 2006; Strange et al., 2014; Tao et al., 2021), including differential innervation of the hippocampus by the SuM (Luo et al., 2025; Soussi et al., 2010). SuM neurons projecting to the dorsal hippocampus (SuM→dHi neurons) can display differing gene expression profiles and, importantly, differing functional roles compared to SuM neurons projecting to the ventral hippocampus (SuM→vHi neurons) (Luo et al., 2025; Soussi et al., 2010). To compare projection patterns of dorsal‐projecting and ventral‐projecting SuM neurons we selectively labelled these cells with a viral vector approach. Specifically we injected a retrograde Flp AAV into either dorsal or ventral DG and a Flp‐dependent AAV into the SuM, allowing selective visualization of retrogradely labelled SuM→dHi and, in separate animals, SuM→vHi neurons and their axon collaterals (Fig. A3). Matching recent reports (Luo et al., 2025) we saw that SuM neurons projecting to the dHi were largely distinct from those projecting to the vHi; retrograde labelling from the dorsal DG resulted in strong labelled SuM innervation of the dHi but only sparse fibres in the vHi (Fig. A3, panels *C*,*G*). Similarly, retrograde labelling from the ventral DG resulted in strong labelled fibres from the SuM in the vHi but only sparse fibres in the dHi (Fig. A3, panels *J*,*K*). Notably, although we targeted retrograde AAV‐Flp to the DG, we consistently saw projection fibres also to CA2 (Fig. A3, panels *D*,*E*,*L*,*M*), including fibres that appear to directly travel between the DG (molecular layer) and neighbouring CA2 (stratum lacunosum moleculare) (Fig. A3, panels *F*,*N*). Therefore, although previous work has shown distinctions between SuM neurons projecting to the dorsal DG and the dorsal CA2 (Borhegyi & Leranth, 1997; Chen et al., 2020; Pan & McNaughton, 2004; Qin et al., 2022; Soussi et al., 2010), there may additionally be some SuM neurons which project to both regions (also see Luo et al., 2025). Importantly, however, we saw generally distinct innervation by the SuM of the dHi and the vHi (Fig. A3, panels *G*,*J*), indicative of largely non‐overlapping populations of SuM→dHi and SuM→vHi cells. Given that we see strong hippocampal innervation of the lateral SuM, where both dorsal‐projecting and ventral‐projecting SuM→Hi neurons are located (Fig. A3, panels *B*,*I*), and because we noted Hi→SuM inhibitory cells originating along the septo‐temporal hippocampal axis (Fig. 1), we next sought to determine not only if the hippocampus directly inhibits SuM neurons that project to the hippocampus but also if there are any specific dorsal–ventral connectivity motifs.

**Figure 4:**
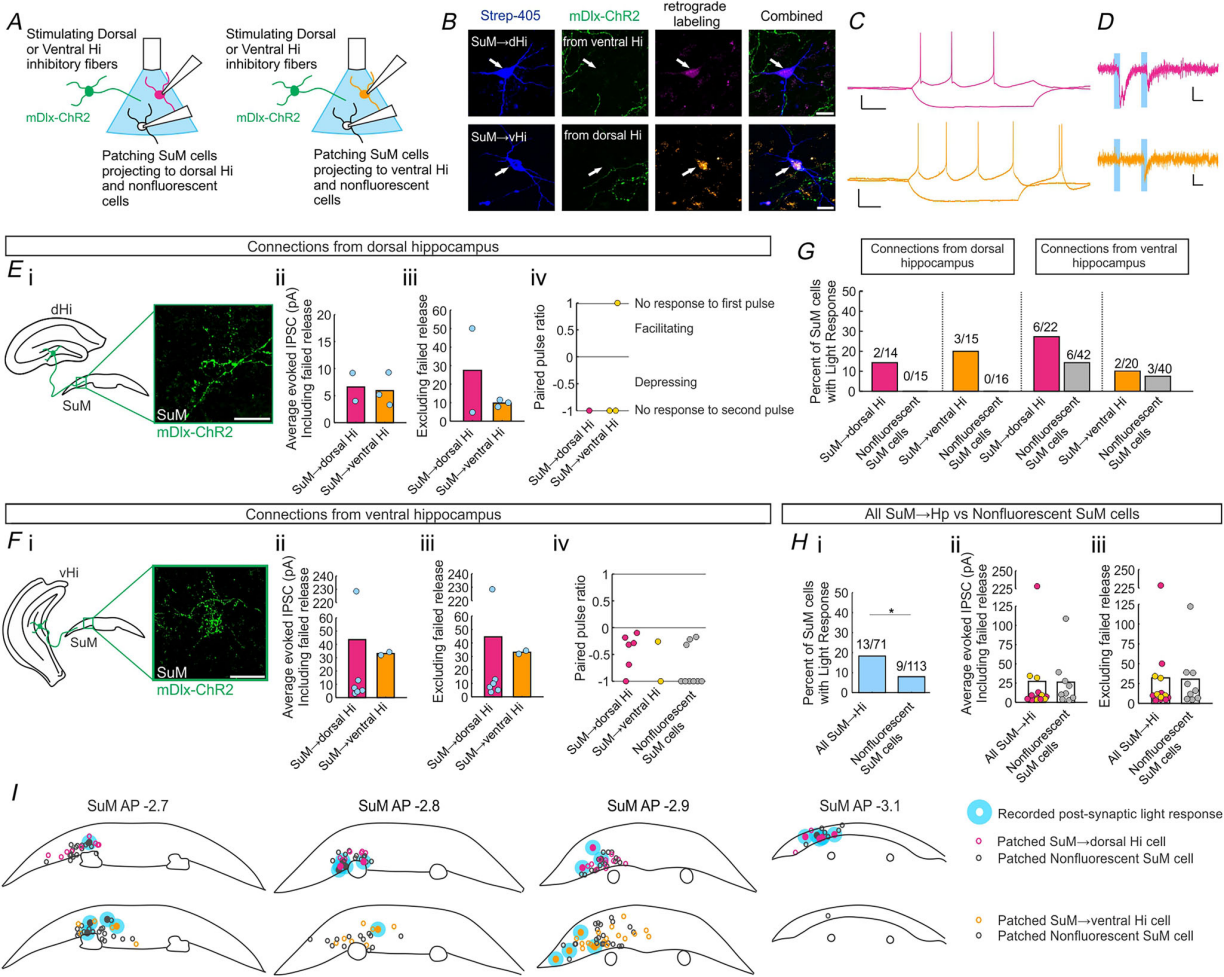
Hippocampal inhibitory cells preferentially target supramammillary area (SuM) neurons projecting back to the hippocampus *A*, schemes for patching SuM neurons while stimulating hippocampal inhibitory projections. ChR2 was expressed in inhibitory cells of either dorsal or ventral hippocampus, and SuM neurons projecting to either dorsal or ventral hippocampus were retrogradely labelled with AAV. *B*, example patched SuM→dHi (top, magenta) and SuM→vHi (bottom, orange) neurons. Cells expressing rg‐mCherry were patched, filled with biocytin, and recovered with streptavidin‐405 (blue). Scale bar: 20 µm. *C*, example voltage traces during −50 pA and +25 pA current injections, corresponding to cells in (*B*). Scale bars: 20 mV, 100 ms. *D*, example average traces of light‐evoked IPSCs, recorded from cells in (*B*). Scale bars: 5 pA, 10 ms. *E*, example image of mDlx‐ChR2^+^ fibres in the SuM, projecting from dorsal hippocampus (i). Scale bar: 50 µm. *E*ii–*E*iii, the average amplitude of evoked inhibitory postsynaptic currents (IPSCs) recorded in response to stimulation of dorsal hippocampal ChR2^+^ fibres. Amplitudes including failed release (ii) and excluding failed release events (iii) are shown. *E*iv, the paired pulse ratio (PPR) of responses recorded in SuM→dHi (magenta) and SuM→vHi (yellow) cells with a 20 ms interpulse interval. One SuM→dHi light responsive cell was not recorded with paired pulse light delivery and is excluded from the PPR. *F*, same as (*E*) but arising from ventral hippocampal inhibitory fibres. *G*, percentage of patched cells with light‐evoked IPSCs. Non‐fluorescent SuM cell columns (grey) correspond to non‐fluorescent cells with an unknown projection identity patched in tissue with the paired virus scheme. *H*i, connectivity rates of fluorescent cells and non‐fluorescent cells pooled across schemes. Direct connectivity from the hippocampus is higher for SuM cells which project back to the hippocampus, relative to cells with an unknown identity (χ^2^ = 4.43, *P* = 0.0353). *H*ii–*H*iii, the average amplitude of evoked IPSCs including failed release (ii) and excluding failed release (iii). *I*, a map of all cells patched, with rg‐mCherry expressed in either SuM cells projecting to dorsal hippocampus (top, magenta) or ventral hippocampus (bottom, orange), and non‐labelled patched cells (grey). Cells with recorded light‐evoked responses are highlighted in blue. *P < 0.05.

**Figure A4:**
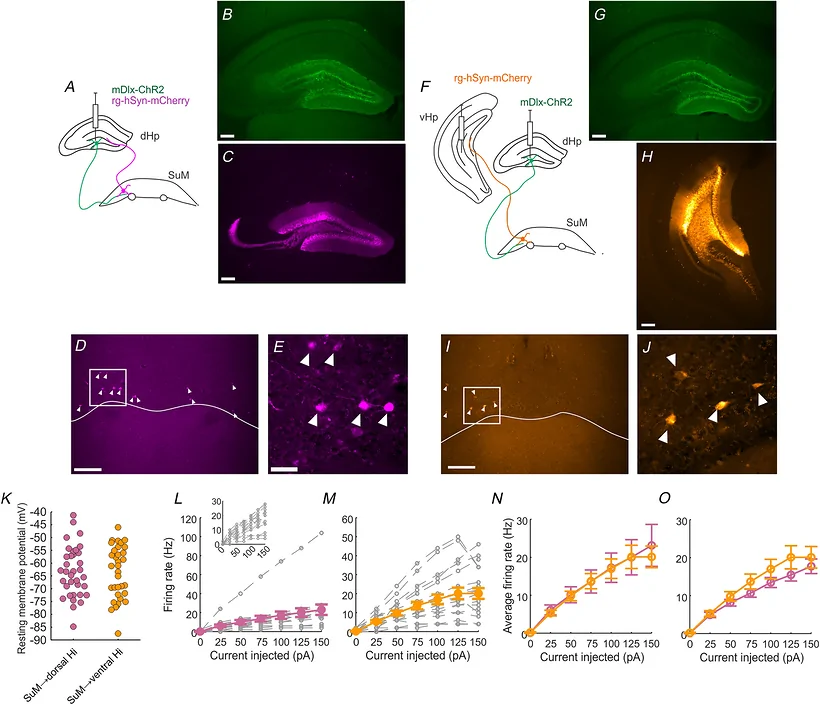
Supramammillary area (SuM) neurons project to dorsal or ventral hippocampus *A*, schematic of injections to express ChR2 in dorsal hippocampal inhibitory neurons and to retrogradely label SuM→dHi neurons. *B*, *C*, example images showing the local hippocampal expression of the mDlx‐ChR2 virus (*B*) and the retrograde‐mCherry virus (*C*). Scale bar: 200 µm. *D*, *E*, example coronal images of retrogradely labelled SuM→dHi neurons. Arrows point to retrogradely labelled neurons. Scale bars: 200 µm (*D*); 50 µm (*E*). (*E*) represents the boxed area in (*D*). *F*–*J*, same as (*A*–*E*), but with retrograde AAV injected into ventral hippocampus to capture SuM→vHi neurons. Images are from 50 µm‐thick fixed tissue. Represented injection schemes were used for electrophysiology experiments presented in Fig. 4. *K*, resting membrane potential recorded at break‐in in SuM→dHi and SuM→vHi neurons. There was no significant difference between populations (−63.3 ± 1.7 mV, *n* = 35 SuM→dHi cells *vs*. −63.9 ± 1.8 mV, *n* = 34 SuM→vHi cells, *P* = 0.86, Mann–Whitney *U* test). *L*, *M*, the evoked firing rate for a subset of SuM→dHi (*n* = 16) (*L*) and SuM→vHi (*n* = 20) (*M*) cells when injecting current steps. Baseline current was injected to maintain the starting membrane voltage at −60 mV. Inset in (*L*) shows the same data with a different *y*‐axis scale. *N*, the average evoked firing rate in SuM→dHi (magenta) and SuM→vHi (orange) neurons. There was no significant difference between the populations (repeated‐measures ANOVA, *F*_(1,34)_ = 0.03, *P* = 0.86). *O*, same as (*N*), but the SuM→dHi population has the maximum outlier removed (i.e. average of data in inset of *L*). There was no significant difference between the populations (repeated‐measures ANOVA, *F*_(1,33)_ = 1.03, *P* = 0.32). Data correspond to experiments shown in Fig. 4.

To this end we injected mice with AAVDJ‐mDlx‐ChR2 into either the dorsal or ventral hippocampus and injected AAVrg‐hSyn‐mCherry into either the dorsal or ventral hippocampus (Fig. A4). This resulted in four separate viral schemes, where either dorsal or ventral hippocampus expressed ChR2 in inhibitory neurons, and either SuM→dHi or SuM→vHi neurons were fluorescently labelled with mCherry (Fig. 4). As with the retrograde‐Flp based labelling approaches described above, rg‐mCherry labelled SuM→dHi or SuM→vHi SuM cells were heavily located in the lateral SuM, near the principle mammillary tract (Fig. A4, panels *D*,*I*); as such, both populations of SuM neurons were primarily in areas with relatively dense hippocampal innervation (Figs 1*F* and 2*H*–*I*). To examine connectivity between hippocampal inhibitory neurons and SuM neurons with projections to the hippocampus we performed whole‐cell patch‐clamp recordings from retrogradely labelled SuM neurons (and for a point of comparison adjacent non‐fluorescent SuM cells), focusing on the ipsilateral SuM. We delivered blue light to activate hippocampal inhibitory fibres and recorded postsynaptic responses.

We found that the hippocampus does indeed directly inhibit SuM neurons which project back to the hippocampus (Fig. 4). Moreover it does so across dorsal–ventral projection patterns, with both dorsal hippocampus and ventral hippocampus inhibiting SuM→dHi and SuM→vHi cells (Fig. 4*E*,*F*). Interestingly there was no significant difference in the connectivity rate across profiles (*dHi innervating SuM→dHi*: 14.3%, 2/14 cells from 18 sections in 9 mice, *vs. dHi innervating SuM→vHi*: 20.0%, 3/15 cells from 8 sections in 5 mice, *P* = 1, Fisher's exact test; *vHi innervating SuM→dHi*: 27.3%, 6/22 cells from 29 sections in 15 mice *vs. vHi innervating SuM→vHi*: 10.0%, 2/20 cells from 22 sections in 12 mice, *P* = 0.243, Fisher's exact test). This indicates that the dorsal hippocampus can influence SuM cells projecting to either the dorsal or the ventral hippocampus, and the ventral hippocampus can influence SuM cells projecting to either the dorsal or the ventral hippocampus, allowing both direct and cross‐talk between subcircuits. Overall, as seen for connections onto neurochemically defined populations, the paired pulse responses were typically depressing (Fig. 4*Eiv*,*Fiv*), indicative of a high release probability. Light‐evoked responses were recorded from neurons in both the anterior and posterior SuM (Fig. 4*I*).

In these experiments we patched both fluorescently labelled SuM→Hi neurons and neighbouring non‐fluorescent SuM cells. Although the projection identity of non‐fluorescent cells is unknown, compared to retrogradely labelled neurons they are less likely to project to the hippocampus, allowing us to determine if hippocampal inhibition is biased towards SuM→Hi neurons over other SuM populations. For all viral vector schemes we consistently found that the proportion of cells receiving hippocampal inhibition was higher for labelled SuM→Hi cells compared to non‐fluorescent cells with unknown projection identity (Fig. 4*G*); overall hippocampal inhibition was significantly biased towards the SuM→Hi population (13/71, 18.3% of SuM→Hi cells collapsed across injection schemes displayed light‐evoked IPSCs *vs*. only 9/113, 8.0% of non‐fluorescent cells, χ^2^ = 4.43, *P* = 0.0353, from 77 sections in 41 mice; Fig. 4*Hi*). When a postsynaptic response was present, the amplitude of IPSCs was not significantly different (absolute value IPSC amplitudes including failed release, SuM→Hi cells: 27.6 ± 17.0 pA [SD: 61.2 pA]; non‐fluorescent cells: 25.6 ± 11.1 pA [SD: 33.4 pA]; *P* = 0.462, MWU; absolute value IPSC amplitudes excluding failed release, SuM→Hi cells: 32.1 ± 16.8 pA [SD: 60.7 pA]; non‐fluorescent cells: 29.9 ± 12.3 pA [SD: 37.0 pA]; *P* = 0.570, MWU; Fig. 4*Hii–iii*).

Overall these data establish that the hippocampus and SuM are bidirectionally connected, whereby the hippocampus can directly inhibit SuM cells providing input to the hippocampus. Interestingly this circuitry connects hippocampal cells across the dorsal–ventral hippocampal axis to SuM→dHi and SuM→vHi neurons, allowing for hippocampal‐SuM circuit cross‐communication.

### Light‐evoked inward and outward postsynaptic currents show similar properties

**Figure A5:**
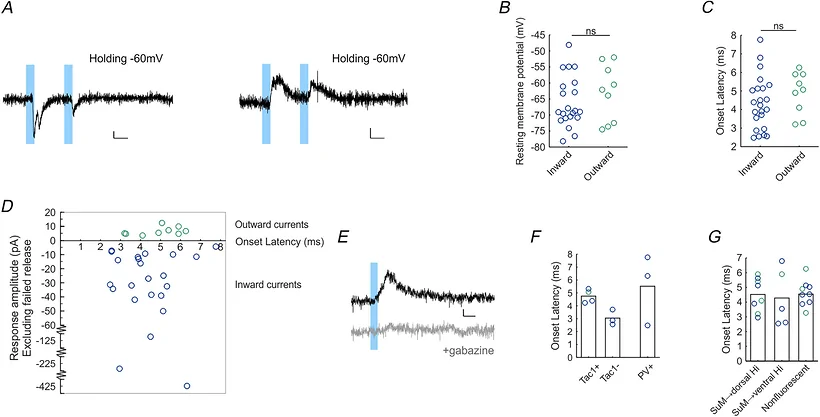
Light‐evoked inward and outward currents show similar onset latencies *A*, example average inward (left) and outward (right) light‐evoked postsynaptic currents. ChR2 was expressed in hippocampal GABAergic neurons, and SuM neurons were recorded in whole‐cell patch configuration, voltage clamped at −60 mV. Scale bars: 5 pA, 10 ms. *B*, resting membrane potential (RMP) recorded after break‐in of a cell, separated by if cells had light‐evoked inward (blue circles) or outward (green circles) current. Data are pooled from all schemes shown in the main text of Figs 3 and 4. RMP is not different between cells with inward responses *vs*. outward responses (−66.0 ± 1.7 mV, *n* = 21 inward responses *vs*. −63.1 ± 2.9 mV, *n* = 9 outward responses, *P* = 0.53, Mann–Whitney *U* test). *C*, onset latency for inward and outward postsynaptic currents. No differences were observed in onset latency of inward *vs*. outward currents (4.3 ± 0.3 ms inward responses *vs*. 4.9 ± 0.4 outward responses, *P* = 0.22, Mann–Whitney *U* test). *D*, the average amplitude of postsynaptic currents plotted against onset latency. Amplitudes were recorded while holding the cell at −60 mV. Onset latency is not correlated with relative amplitude of responses (Pearson's *R* = −0.08, *n* = 31 responses, *P* = 0.68). *E*, example trace of an outward current (black trace) that is eliminated by gabazine (grey trace). Holding voltage is −45 mV for this trace to visualize an increased amplitude of the outward current. The direction of this response was still outward while holding at −60 mV. *F*, onset latency is classified by cell type. Data are from experiments shown in Fig. 3. *G*, same as (*F*) but for SuM→dHi, SuM→vHi and non‐fluorescent SuM cells. Non‐fluorescent cell data pool all patched SuM cells that had no viral labelling, recorded in both the fluorescent SuM→dHi and SuM→vHi virus schemes. Data are from experiments shown in Fig. 4.

All *ex vivo* slice electrophysiology experiments were conducted by optogenetically activating hippocampal projection fibres while recording from SuM neurons using a high chloride intracellular solution, allowing for recording inward GABA_A_‐mediated currents while holding the cell membrane potential near rest at −60 mV. Interestingly even while holding at −60 mV we could observe both the expected inward light‐evoked postsynaptic currents (PSCs) and, surprisingly, occasional *outward* light‐evoked PSCs (Fig. A5). An outward current in these recording conditions would typically be attributed to GABA_B_ receptor activation of a potassium (e.g. GIRK) channel (Christenson Wick et al., 2019; Dascal, 1997). However GABA_B_‐mediated currents typically have slow kinetics on the scale of 100–200 ms (Bettler et al., 2004; Sodickson & Bean, 1996), and the observed light‐evoked outward currents showed short latencies, with comparable onset latencies to those observed for light‐evoked inward currents (inward current onset latency, 4.3 ± 0.3 ms [SD: 1.4 ms], *n* = 23 responses *vs*. outward current onset latency, 4.9 ± 0.4 ms [SD: 1.1 ms], *n* = 9 responses, *P* = 0.224, MWU) (Fig. A5, panels *C*,*D*). Additionally both inward and outward responses were sensitive to the GABA_A_ receptor antagonist gabazine (16/16 light‐evoked responses were eliminated by gabazine application; 11 inward currents, 5 outward currents) (Fig. A5, panel *E*), indicating that these are GABA_A_‐mediated inhibitory responses. Therefore outward responses may instead be potentially attributed to local differences in relative chloride concentrations, whether due to experimental considerations (such as insufficient dispersion of the high chloride intracellular recording solution to distal dendrites – despite the ability to fill the neurons with biocytin contained within the intracellular solution) or, more interestingly, potentially biological differences in internal or external influences on local chloride concentrations across connections (Glykys et al., 2014; Normoyle et al., 2025). Notably, regardless of the cause of the outward GABA_A_‐mediated currents, all responses were consistent with the hippocampus providing inhibition, not excitation, to the SuM.

### Hippocampal inhibitory neurons preferentially target burst‐wave firing neurons of the SuM

**Figure 5:**
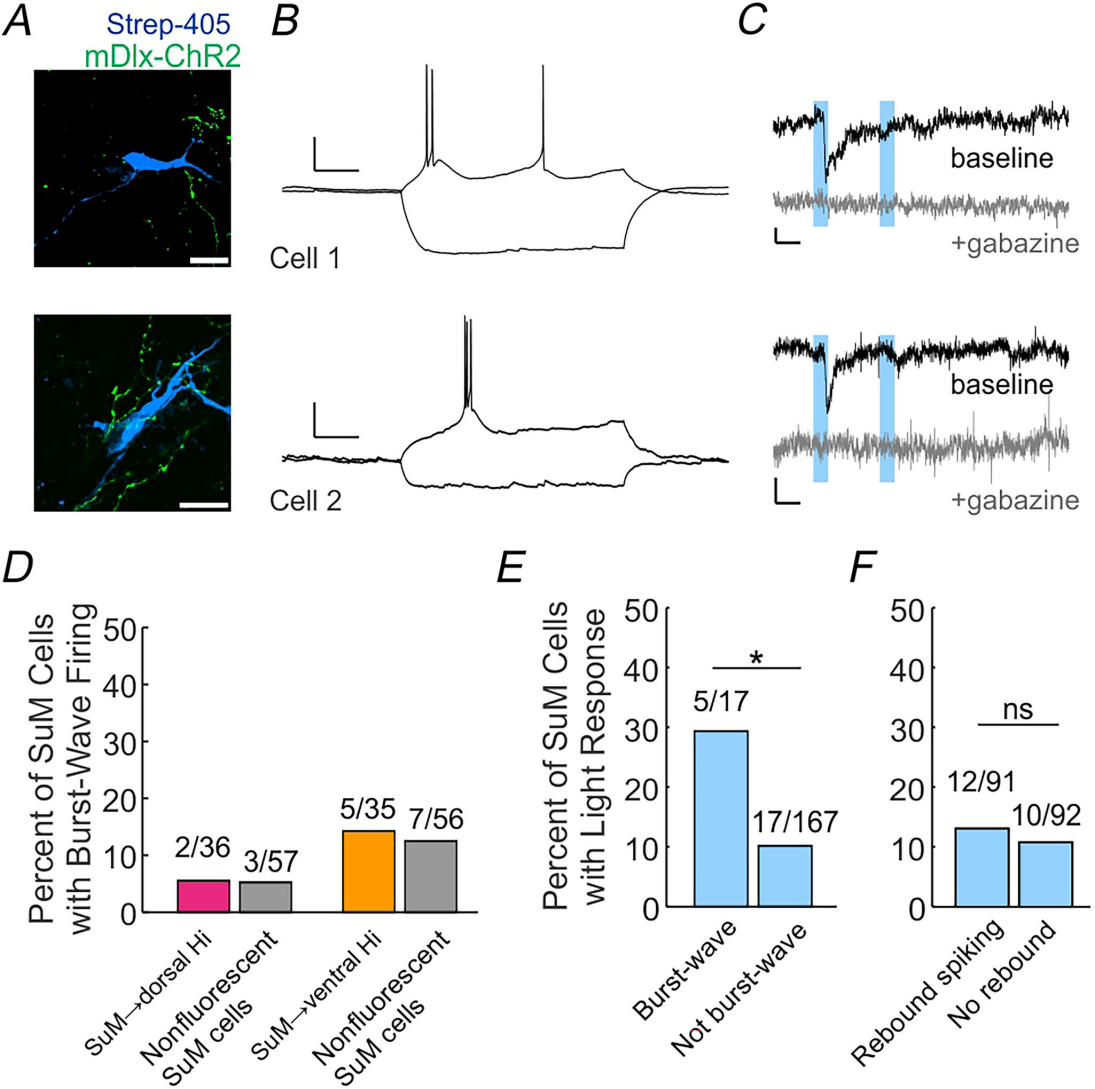
Hippocampal inhibitory neurons preferentially target burst‐wave neurons of the supramammillary area (SuM) *A*, SuM cells were patched and light‐evoked inhibitory postsynaptic currents (IPSCs) recorded, with mDlx‐ChR2 expressed in dorsal or ventral hippocampal fibres. Cells were filled with biocytin and recovered with streptavidin‐405 (blue). Data correspond to schemes presented in Fig. 4. Scale bar: 20 µm. *B*, example voltage traces from patched SuM cells with −100 pA and +25 pA injected current (top) or −100 pA and +75 pA injected current (bottom). Each cell shows a burst‐wave firing pattern near threshold, with a cluster of two or more spikes with short interspike intervals (burst) followed by a slow afterhyperpolarization (wave). Scale bars: 20 mV, 100 ms. *C*, example average traces of light‐evoked IPSCs corresponding to cells in (*A*). Gabazine abolished light‐evoked IPSCs (grey traces). Scale bars: 5 pA, 10 ms. *D*, percentage of rg‐mCherry and non‐fluorescent patched cells displaying a burst‐wave firing pattern by injection scheme. Burst‐wave cells can project to the dorsal (magenta) or ventral (orange) hippocampus. *E*, the percentage of burst‐wave firing cells with light‐evoked IPSCs compared to all other patched cells. There is a higher probability for burst‐wave cells to receive direct hippocampal inhibition compared to non‐burst‐wave firing cells (χ^2^ = 5.4, *P* = 0.0199). *F*, same as (*E*) but for rebound spiking cells. There was no significant difference in innervation of rebound spiking cells *vs*. non–rebound spiking cells (χ^2^ = 0.23, *P* = 0.630). *P < 0.05. ns, not significant.

In patching SuM cells we discovered a diversity of firing types, including a small subset of cells that showed a unique near‐threshold burst‐wave firing pattern, characterized by a short interspike interval (first interspike interval: 9.98 ± 1.30 ms [SD: 6.11 ms], *n* = 22 cells) followed by a slow‐wave afterhyperpolarization (Venkadesh et al., 2019) (22/290 cells from 116 sections in 65 mice, 7.6%, of all patched SuM cells displayed a burst‐wave firing pattern) (Fig. 5). This suggests a likely distinct functional role for these cells and is especially interesting as previous *in vivo* work has shown that burst‐firing neurons of the SuM are active during hippocampal sharp wave ripples (Caneo et al., 2026; Vicente et al., 2020), and that SuM cells tend to burst‐fire with a theta‐phase preference (Caneo et al., 2026; Cissé et al., 2026; Kirk, 1998; Kirk & McNaughton, 1991; Kocsis & Vertes, 1994).

Although we found that both Tac1^+^ and Tac1^−^ cells recorded from lateral SuM could display rebound spiking (Fig. A2, panels *A*–*C*), and rebound spiking is reported in Tac1^+^ cells (Farrell et al., 2021), the rebound firing we observed in most Tac1 cells was a distinct firing pattern compared to burst‐wave firing (compare burst‐wave examples in Fig. 5 to the example rebound in Figs 3*E* and A2, panels *A*,*B*). Regarding burst‐wave firing observed while injecting current to depolarize cells, only a very small percentage of Tac1^+^ and Tac1^−^ cells showed burst‐wave firing, and this was similar between Tac1^+^ and Tac1^−^ cells (Tac1^+^: 1/22, 4.5%, of patched cells displayed burst‐wave firing; Tac1^−^: 1/16, 6.3%, *P* = 1, Fisher's exact test). No SuM PV^+^ cells patched displayed a burst‐wave firing pattern (0/13 PV^+^ cells). NOS‐expressing cells are also present in the SuM and are reported to impact wakefulness (Pedersen et al., 2017). To determine if NOS^+^ SuM cells display burst‐wave firing patterns we injected NOS‐Cre mice with AAV‐FLEX‐tdTomato in the SuM and then patched fluorescently labelled cells. Similar to populations defined by Tac1 expression, only a very small proportion of NOS^+^ SuM cells displayed a burst‐wave firing pattern (NOS^+^: 1/17, 5.9%, of patched cell displayed burst‐wave firing). Therefore of the neurochemical markers examined none were predictive of a burst‐wave firing pattern.

Cells with a burst‐wave firing pattern were observed in both SuM→dHi and SuM→vHi cells. For SuM→dHi cells this percentage was again very low (SuM→dHi: 2/36 patched cells, 5.5%) (Fig. 5*D*). A notably larger percentage of SuM→vHi cells displayed a burst‐wave firing pattern, but this remained well below a majority (SuM→vHi: 5/35 cells, 14.3% burst‐wave firing) (Fig. 5*D*). In addition to the hippocampus the SuM has projections to the medial septum (MS) (Farrell et al., 2021; Liang et al., 2023; Luo et al., 2025; Vertes & McKenna, 2000). To determine whether burst‐wave firing cells might be enriched in SuM cells projecting to the MS (SuM→MS cells) we completed additional experiments recording from SuM→MS cells labelled after retrograde AAV injection into the MS. However none of these displayed a burst‐wave firing pattern (0/11 patched SuM→MS cells), making it unlikely that burst‐wave SuM cells represent SuM→MS cells. Overall these experiments suggest that burst‐wave cells make up a small population of SuM cells not defined by any of the neurochemical markers examined and with the greatest abundance within the SuM→vHi cell population.

In schemes with mDlx‐ChR2 expressed in either dorsal or ventral hippocampus (see Fig. 4) we found that burst‐wave cells were preferentially targeted by hippocampal inhibition compared to non‐burst‐wave firing cells (5/17, 29.4% of burst‐wave cells displayed light‐evoked IPSCs; *vs* 17/167, 10.2% of non‐burst‐wave cells displayed a light‐evoked IPSC; χ^2^ = 5.4, *P* = 0.0199) (Fig. 5*E*). Therefore, although less than 10% of SuM cells show a burst‐wave firing pattern, burst‐wave cells are preferentially innervated by hippocampal inhibitory inputs. As noted earlier, rebound firing represents another firing type noted in some patched SuM cells, in which cells fire action potentials after release from a hyperpolarizing current step. In contrast to burst‐wave firing, we found that rebound spiking was not predictive of hippocampal innervation. This was true both for schemes where mDlx‐ChR2 was expressed in either dorsal or ventral hippocampus (12/91, 13.2% of rebound firing cells displayed light‐evoked IPSCs; *vs*. 10/92, 10.9% of non‐rebound firing cells displayed light‐evoked IPSCs; χ^2^ = 0.23, *P* = 0.630) (Fig. 5*F*) and for schemes with mDlx‐ChR2 expressed across the hippocampus in Tac1‐Cre mice (5/19, 26.3% of rebound firing cells displayed light‐evoked IPSCs; *vs*. 2/19, 10.5% of non‐rebound firing cells; *P* = 0.405, Fisher's exact test) (Fig. A2, panel *D*). This suggests that burst‐wave firing represents a uniquely informative firing pattern with regard to likelihood of hippocampal inhibitory innervation.

## Discussion

Recent years have shown increasing interest in the SuM, its projections to the hippocampus, and its influence on hippocampal‐dependent memory and oscillations (Billwiller et al., 2020; Caneo et al., 2026; Chen et al., 2020; Farrell et al., 2021; Y. Li et al., 2020; Luo et al., 2025; Luppi et al., 2024; Qin et al., 2022; Robert et al., 2021; Vicente et al., 2020; Young et al., 2024). Our work highlights an understudied connection in the opposite direction – specifically hippocampal input to the SuM. We find that this connection is inhibitory and arises from both SST^+^ and NOS^+^ GABAergic neurons, from across hippocampal subregions and across the dorsal–ventral hippocampal axis. Hippocampal input to the SuM equally targets the anterior and posterior SuM but provides only sparse innervation to the medial SuM, with much denser innervation to lateral areas in which SuM→Hi neurons are preferentially located. We find that Hi→SuM cells inhibit functionally and neurochemically diverse SuM cells, while preferentially targeting burst‐wave firing and, importantly, SuM→Hi neurons. This connectivity therefore provides a means for the hippocampus to broadly influence the SuM, including direct inhibitory control of SuM input to the hippocampus.

Hi→SuM cells are not localized to one hippocampal region but are instead found in all hippocampal CA regions, especially within the oriens, and in the DG hilus. They include both long‐range inhibitory NOS^+^ cells (i.e. LINCs) (Christenson Wick et al., 2019) and SST^+^ cells (Kinney et al., 2025). We find that Hi→SuM cells include both mixed NOS^+^/SST^+^ cells and cells that exclusively express NOS or SST. Additionally although both LINCs and SST^+^ hippocampal cells project to the SuM, there are slight differences in the projection patterns of hippocampal SST^+^ and NOS^+^ cells within the SuM. Ventral hippocampal SST^+^ cells in particular provide strong, ipsilateral focused, input to the SuM. Interestingly both LINCs and SST^+^ hippocampal cells provide only sparse innervation to the medial SuM. Although there are no firm borders between the medial and lateral SuM, the medial SuM contains different cell populations and is associated with different functions compared to the more lateral SuM (Allen Gonzalo‐Ruiz et al., 1992; Institute & Zeng, 2023; Kesner et al., 2023; Pan & McNaughton, 2004; Soussi et al., 2010; Yao et al., 2023). For example, tyrosine hydroxylase‐expressing cells in the medial SuM project to the dorsomedial hypothalamus and are associated with diet and metabolic regulation (Plaisier et al., 2020; Swanson, 1982; Zhang et al., 2023). The lack of hippocampal fibres in the medial SuM suggests that the hippocampus is unlikely to provide direct influence on these neurons and associated functions. In contrast, strong hippocampal fibres were seen in areas of the SuM with high concentrations of SuM→Hi neurons, suggesting that tuning of SuM inputs to the hippocampus is a key feature of Hi→SuM cells.

Like Hi→SuM cells, hippocampal‐septal cells are GABAergic projection neurons, are preferentially located in the oriens (Alonso & Köhler, 1982; Jinno & Kosaka, 2002) and express SST (Jinno, 2009; Jinno & Kosaka, 2002; Jinno et al., 2007; Yuan et al., 2017). As we were unable to label Hi→SuM cells through retrograde AAV approaches (see Methods) we were not able to determine Hi→SuM collaterals to other regions, including the MS. Recent work suggests that at least a subset of SST^+^ hippocampal‐septal cells have collaterals to the SuM (Kinney et al., 2025), and LINCs collectively project to several downstream regions, including the tenia tecta and, to a lesser degree, the MS. Notably, approximately 40% of hippocampal‐septal cells express M2R (Jinno, 2009; Jinno et al., 2007), a marker characteristic of TORO cells (which have projections to many regions, including the MS) (Szabo et al., 2022). Almost all Hi→SuM cells were M2R immunonegative and are therefore likely neither TORO cells nor M2R^+^ hippocampal‐septal cells.

Although long‐range inhibitory cells frequently target inhibitory cells in the target region, and thus play a net disinhibitory circuit role (Basu et al., 2016; Freund & Antal, 1988; Freund & Gulyás, 1991; Malik et al., 2022; Melzer et al., 2012; Nieh et al., 2016; Toth et al., 1993), we find that Hi→SuM cells do not show this preference. Instead our data suggest that Hi→SuM cells preferentially target SuM neurons that project to the hippocampus (which are either glutamatergic or dual glutamatergic/GABAergic) (Billwiller et al., 2020; Boulland et al., 2009; Chen et al., 2020; Hashimotodani et al., 2018; Jiang et al., 2024; M. Li et al., 2023; Y. Li et al., 2020; Robert et al., 2021; Soussi et al., 2010). Notably we also found that Hi→SuM cells can inhibit fast‐spiking PV+ SuM cells, but, in our hands, this was not a higher rate than other neurochemically defined SuM populations. Unlike in neocortex or hippocampus, PV^+^ cells in the hypothalamus may be glutamatergic or GABAergic (Allen Institute & Zeng, 2023; L. Li et al., 2025; Yao et al., 2023), and the neurotransmitter profile of PV^+^ cells in the SuM is unclear. However per the Allen Institute's online Merfish data (Allen Institute & Zeng, 2023; Yao et al., 2023) the cells with the highest levels of PV expression in the SuM are GABAergic. If SuM PV^+^ cells are GABAergic and target local SuM neurons, Hi→SuM cells may additionally play a disinhibitory role. However, as Hi→SuM cells do not appear to preferentially target PV^+^ cells, disinhibition is unlikely the primary role. Instead we find that hippocampal input to the SuM preferentially targets SuM→Hi cells, thereby providing a means for direct hippocampal tuning of SuM input to the hippocampus.

Postsynaptic targets of Hi→SuM cells are neurochemically diverse and include both Tac1^+^ and Tac1^−^ cells, as well as PV^+^ cells. Both Tac1^+^ and Tac1^−^ cells include projection populations that target many regions, including the hippocampus (Farrell et al., 2021). Tac1^+^ SuM neurons’ activity reflects speed and locomotion, whereas Tac1^−^ cells with projections to the medial septum influence hippocampal spike timing and theta oscillations (Farrell et al., 2021). This suggests that Hi→SuM cells can inhibit SuM neurons with a range of physiological roles. As an interesting side note we saw rebound firing in both Tac1^+^ and Tac1^−^ cells (36.4% of Tac1^+^ cells and 68.8% of Tac1^−^ cells showed rebound firing after release from a hyperpolarization step). In contrast, previous work reported rebound firing as a distinguishing feature specific to Tac1^+^ cells (Farrell et al., 2021). All of our recordings were focused in lateral SuM to capture cells in regions with dense Hi→SuM projection fibres, and we used Tac1xTOM mice to genetically label and record from Tac1^+^ cells, with non‐labelled cells serving as our Tac1^−^ population. The previous study instead used a more restrictive viral vector approach and examined Tac1^+^ cells broadly but specifically Tac1^−^ cells with projections to the MS (Farrell et al., 2021). Therefore it is possible that a lack of rebound firing is specific to this subset of Tac1^−^ cells. Notably we saw no significant difference in the proportion of cells receiving hippocampal inhibition when comparing rebound spiking *vs*. non–rebound spiking populations.

Consistent with recent reports (Luo et al., 2025) we confirmed that SuM projections to the dDG and vDG are mediated by largely separate populations in the mouse (Fig. A3), although these cell populations do not exhibit the same spatial segregation within the SuM as reported in the rat (Soussi et al., 2010) (Fig. A3). When separating dorsal and ventral hippocampal inhibitory neurons to determine if they target SuM neurons that project to dorsal or ventral hippocampus, we see that there is mixed communication across the dorsal–ventral axis. That is to say that dorsal hippocampal inhibitory neurons target both SuM→vHi and SuM→dHi cells, and the same is true for ventral hippocampal inhibitory neurons. Previous literature indicates that SuM neurons projecting to the ventral dentate are a glutamatergic population (Soussi et al., 2010), and SuM→dHi can be either glutamatergic or dual glutamatergic/GABAergic (Kesner et al., 2023). More specifically, the dorsal DG is targeted by a dual glutamatergic/GABAergic population (Ajibola et al., 2021; Billwiller et al., 2020; Boulland et al., 2009; Chen et al., 2020; Hashimotodani et al., 2018; Hirai et al., 2022, 2024; Y. Li et al., 2020; Pedersen et al., 2017; Soussi et al., 2010), whereas the dorsal CA regions (Escobedo et al., 2024; Jiang et al., 2024; M. Li et al., 2023), including dCA2 (Chen et al., 2020; Robert et al., 2021; Soussi et al., 2010), are believed to be targeted by a solely glutamatergic population. There are some reports that GABAergic (i.e. VGAT‐expressing) SuM neurons may also project to the CA2 (Chen et al., 2020; Y. Li et al., 2020; Pedersen et al., 2017), and recently Luo et al. found fibres in the dorsal CA2 after retrogradely labelling SuM cells from dorsal DG (Luo et al., 2025). However, given that extensive previous literature reports that dDG and dCA2 are innervated by distinct populations of SuM neurons in both rat (Borhegyi & Leranth, 1997; Pan & McNaughton, 2004; Soussi et al., 2010) and mouse (Chen et al., 2020; Qin et al., 2022), we were still surprised that our intersectional AAV tracing work consistently showed strong innervation of both dDG and dCA2 concomitantly. This is despite our AAV coordinates targeting dDG specifically and our other retrograde AAV schemes showing fairly selective spread of virus within the DG (see Fig. A4). Although the potential for spillover can never be completely eliminated, we notably see labelled fibres travelling between dDG and CA2 (Fig. A3). We therefore tentatively conclude that there may be populations of SuM cells which target either the dDG or the dCA2, but not both, and a population of SuM cells which target both the dDG and dCA2. Importantly, regardless of potential collaterals to other regions we were able to selectively label SuM→dHi *vs*. SuM→vHi populations, and both of these received inhibitory hippocampal inputs. We found that the pooled populations of SuM→Hi cells were preferentially targeted by hippocampal inhibition over the unlabelled, non‐fluorescent SuM cells of unknown projection identity, which received sparse innervation. Of note, these concomitantly patched non‐fluorescent cells may include hippocampal‐projecting SuM cells that were not captured by the retrograde AAV, either due to the spread of the virus injection not capturing this hippocampal target or due to cell‐type variability in transduction with the AAV retrograde serotype (Tervo et al., 2016). Thus the sparsely innervated non‐fluorescent SuM cells may also include unlabelled SuM→Hi cells.

SuM→dHi neurons impact hippocampal oscillations (Billwiller et al., 2020; Farrell et al., 2021; Ito et al., 2018; Kirk, 1998; Kirk & McNaughton, 1991; Kocsis & Vertes, 1994; Qin et al., 2022), play a role in both sleep and locomotion (Billwiller et al., 2020; Farrell et al., 2021; Qin et al., 2022; Renouard et al., 2015) and impact spatial and, via projections to CA2, social memory (Chen et al., 2020; Y. Li et al., 2020; Luo et al., 2025; Qin et al., 2022). In contrast SuM→vHi neurons play a larger role in more affective behaviours, including sucrose preference and mobility during tail suspension (Luo et al., 2025). Hi→SuM cells are positioned to influence SuM projections to either dorsal or ventral hippocampus and, therefore, also to shape functions associated with those populations, including memory and active coping strategies (Escobedo et al., 2024; Kesner et al., 2023; Luo et al., 2025). This is true for both dorsal and ventrally located Hi→SuM neurons. Notably hippocampal input to the SuM is therefore not merely a form of feedback inhibition but can also provide cross‐talk between supramammillary‐hippocampal subcircuits, with ventral hippocampus influencing SuM cells projecting to the dorsal hippocampus, and vice versa.

In addition to preferentially targeting SuM→Hi cells, we find that Hi→SuM inputs preferentially target burst‐wave firing neurons of the SuM. Although Hi→SuM cells provide input to SuM neurons with diverse firing properties, and only a small fraction of all patched SuM cells displayed a burst‐wave firing pattern, burst‐wave cells were disproportionally targeted by Hi→SuM input. Indeed in our experiments burst‐wave firing cells were thrice as likely to receive hippocampal input than non‐burst‐wave cells. *In vivo* burst firing SuM neurons are associated with hippocampal sharp‐wave ripples (Caneo et al., 2026; Vicente et al., 2020) and theta‐phase activity (Caneo et al., 2026; Cissé et al., 2026; Kirk, 1998; Kirk & McNaughton, 1991; Kocsis & Vertes, 1994), and it will be important for future work to determine if these cells characterized as ‘burst firing’ *in vivo* correspond to the burst‐wave cells preferentially targeted by hippocampal input. We did not find a defining neurochemical identity of burst‐wave firing cells, with neither Tac1, PV nor NOS‐expression being informative. However the SuM contains a wealth of neurochemically diverse cell populations; a specific neurochemical identity for burst‐wave SuM cells remains a possibility. Whatever their neurochemical identity, their distinct firing pattern and apparent preferential input from hippocampal inhibitory neurons suggest a unique functional role for SuM burst‐wave cells.

We show here that hippocampal inhibitory neurons innervate the SuM, and therefore hippocampus‐supramammillary interactions are not unidirectionally limited to the SuM impacting the hippocampus. This inhibitory connection comes from multiple hippocampal neuron types (including LINCs and SST^+^ cells), from multiple regions (including the hilus, CA3, CA2 and CA1) and from across the septo‐temporal axis of the hippocampus. This inhibitory connection also targets a variety of SuM neurons while preferentially targeting SuM neurons with projections back to the hippocampus. Therefore hippocampal‐supramammillary interactions are not unidirectional but instead bidirectional; via Hi→SuM inhibitory cells, the hippocampus can directly influence the SuM, including its influence on the hippocampus itself.

## Materials and methods

### Ethical approval

All experimental protocols were approved by the University of Minnesota's Institutional Animal Care and Use Committee (protocol #2310‐41437A). These protocols comply with the Public Health Service Policy on Humane Care and Use of Laboratory Animals and the National Research Council Guide for the Care and Use of Laboratory Animals.

### Animals

Adult male and female mice older than P45 were used in all experiments. C57Bl/6J founder mice were purchased from Jackson Laboratory (Bar Harbor, ME, USA) (IMSR_JAX:000664) and subsequently bred in house. These mice were used for fluorogold tracing experiments and retrograde viral tracing experiments, including slice electrophysiology experiments with retrogradely labelled SuM cells projecting to the hippocampus. Transgenic mice expressing Cre in somatostatin^+^ cells (SST‐Cre) (IMSR_JAX:013044, *SST^tm2.1(cre)Zjh^*/J) (Taniguchi et al., 2011), NOS^+^ cells (NOS‐Cre) (IMSR_JAX:017526, # B6.129‐*Nos1^tm1(cre)Mgmj^*/J) (Leshan et al., 2012), PV^+^ cells (PV‐Cre) (IMSR_JAX:008069, B6;129P2‐*Pvalb^tm1(cre)Arbr^*/J) (Hippenmeyer et al., 2005), GAD2^+^ cells (GAD‐Cre; used for some retrograde viral testing, see below) (IMSR_JAX:28867, B6J.Cg‐*Gad2^tm2(cre)Zjh^*/MwarJ) (Hwang et al., 2017) or Tac1^+^ cells (Tac1‐Cre) (IMSR_JAX:021877, B6;129S‐*Tac1^tm1.1(cre)Hze^*/J) (Harris et al., 2014) were purchased from Jackson Laboratory and subsequently bred in house for experiments. GAD‐Cre founders, SST‐Cre founders and Tac1‐Cre founders were crossed with C57Bl/6J mice and maintained as hemizygotes. NOS‐Cre and PV‐Cre mice were maintained as homozygotes. Transgenic Cre‐reporter founder mice expressing Cre‐dependent tdTomato (TOM) (IMSR_JAX:007909, B6.Cg‐*Gt(ROSA)26Sor^tm9(CAG‐tdTomato)Hze^*/J) (Madisen et al., 2010) were also purchased from Jackson Laboratory and subsequently maintained as homozygotes. Tac1‐Cre and PV‐Cre mice were crossed with TOM homozygous mice to generate Tac1xTOM and PVxTOM mice, respectively.

All mice were group housed in the animal facility at the University of Minnesota, with *ad libitum* access to food and water, and a 14:10 light–dark cycle. Before tissue harvest for all experiments mice were deeply anaesthetized with 5% isoflurane, assessed for anaesthesia level via toe pinch and killed via guillotine.

### Stereotaxic surgeries

All injections were given using a Nanoject pressure injector injecting at 40 nL/min. Mice were anaesthetized in an induction chamber with 2%–3% isoflurane, shaved and then injected with carprofen (subcutaneous 10–20 mg/kg) and bupivacaine (subcutaneous at incision site, <5 mg/kg) to alleviate pain prior to incision. Mice were then placed in ear bars in a stereotaxic set‐up. Surgical procedures were performed under 1%–3% isoflurane anaesthesia, and depth of anaesthesia was monitored via toe pinch and breathing pattern (Armstrong et al., 2013; Christenson Wick et al., 2019; Krook‐Magnuson et al., 2013). After surgery mice were monitored for 3 days to observe recovery. During this time the posture, behaviour and grooming of each mouse was scored to assess any signs of pain or distress. If signs of distress were observed, a dose of carprofen was administered (subcutaneous 10–20 mg/kg).

#### Fluorogold injections

Mice were injected with 10 nL of fluorogold (FG; 4% in saline; Fluorochrome LLC; cat#52‐9400) into the supramammillary area (SuM) using angled stereotaxic co‐ordinates that avoided passing through the hippocampus (15° angle tilted back towards posterior brain; 3.75 mm posterior, 0.5 mm left of bregma, 5 mm down angled axis). This volume was used to maximize specificity to the SuM. Additionally to keep the injection tract clean of fluorogold we loaded 1–2 nL of mineral oil into the glass tip of the pressure injector. Brains were harvested 1 week post‐injection and were fixed in 4% paraformaldehyde (PFA) overnight. Only tissue from animals with correctly targeted injections was used.

#### Virus injections

To quantify hippocampal terminals in the SuM, NOS‐Cre and SST‐Cre mice were injected with 200 nL of either AAV1‐phSyn1(S)‐FLEX‐tdTomato‐T2A‐SypEGFP‐WPRE (phSyn1‐FLEX‐SypEGFP, Addgene viral prep # 51509‐AAV1, generously donated by Hongkui Zeng (Oh et al., 2014), titre 1.6×10^13^ vg/mL) or AAVDJ‐mDlx‐dio‐mScarlet‐T2A‐SYPEGFP (Addgene plasmid # 185696, p226‐AAV‐dlx‐dio‐mScarlet‐T2A‐SYPEGFP, generously donated by Lynette Lim; packaged by the UMN Viral Vector and Cloning Core, titre 5.35×10^12^ vg/mL) into either dorsal hippocampus (1.9 mm posterior, 1 mm left, 2 mm ventral relative to bregma) (phSyn1‐FLEX‐SypEGFP: *n* = 1 female, 1 male SST‐Cre mice; *n* = 2 male NOS‐Cre mice; mDlx‐FLEX‐SypEGFP: *n* = 1 female, 1 male SST‐Cre mice; *n* = 2 female NOS‐Cre mice) or ventral hippocampus (3.4 mm posterior, 2.6 mm left, 3.8 mm ventral relative to bregma) (phSyn1‐FLEX‐SypEGFP: *n* = 1 female, 1 male SST‐Cre mice; *n* = 2 female NOS‐Cre mice; mDlx‐FLEX‐SypEGFP: *n* = 1 female, 1 male SST‐Cre mice; *n* = 2 male NOS‐Cre mice). Virus was allowed to incubate for 3–4 weeks before the tissue was harvested. Only mice with on‐target hippocampal expression were used for experiments.

For intersectional tracing of SuM projections to either dorsal or ventral hippocampus, mice were injected with 100 nL of AAVrg‐Ef1a‐Flpo (Addgene viral prep # 55637‐AAVrg, generously donated by Karl Deisseroth (Fenno et al., 2014), titre 1.7×10^12^ vg/mL) into either dorsal (1.9 mm posterior, 1 mm left, 2 mm ventral relative to bregma) or ventral hippocampus (3.4 mm posterior, 2.6 mm left, 3.8 mm ventral relative to bregma) and 200 nL of Flp‐dependent vector AAV8‐Ef1a‐fDIO‐mCherry (Addgene viral prep # 55641‐AAV9, generously donated by Karl Deisseroth (Fenno et al., 2014), titre 2.0×10^11^ vg/mL) or AAV9‐Ef1a‐fDIO‐EYFP (Addgene viral prep # 114471‐AAV9, generously donated by Karl Deisseroth, titre 2.2×10^12^ vg/mL) into the SuM (15° angle tilted back towards posterior brain; 3.75 mm posterior, 0.5 mm left of bregma, 5 mm down the angled axis). Virus was allowed to incubate for at least 3 weeks before the tissue was harvested.

For slice electrophysiology with optogenetics mice were injected with AAVDJ‐mDlx‐ChR2‐EYFP‐mGluR2‐PEST‐ATE (mDlx‐ChR2), a terminal‐targeted ChR2 (sequence kindly provided by Ayako M. Watabe and Toshihisa Ohtsuka (Hamada et al., 2021)) prepared using an mDlx enhancer and packaged by the UMN Viral Vector and Cloning Core (titre 1.4×10^14^ vg/mL). For experiments with mDlx‐ChR2 expressed broadly across the hippocampus mice were injected with the following scheme: 150 nL at 3.2 mm posterior and 2.6 mm left of bregma, in 50 nL increments at 4.2, 3.4 and 2.6 mm ventral to bregma; 150 nL at 2.8 mm posterior and 3.0 mm left of bregma, in 50 nL increments at 4.2, 3.4 and 2.6 mm ventral to bregma; and 100 nL at 1.9 mm posterior, 1.0 mm left and 2.0 mm ventral to bregma (*n* = 7 Tac1‐Cre mice, 3 females and 4 males; *n* = 2 PV‐Cre mice, 1 female and 1 male). Other experiments selectively expressed mDlx‐ChR2 into either dorsal (*n* = 14 Bl6 mice; 4 females, 10 males) or ventral (*n* = 27 Bl6 mice; 12 females, 15 males) hippocampus. SuM neurons with projections to the hippocampus were identified with injection of AAVrg‐hSyn‐mCherry (rg‐mCherry) (Addgene viral prep # 114472‐AAVrg, generously donated by Karl Deisseroth, titre 1.9×10^12^ vg/mL) into the corresponding region – dorsal hippocampus: 1.9 mm posterior, 1.0 mm left and 2.0 mm ventral to bregma; ventral hippocampus: 3.3–3.4 mm posterior, 2.6 mm left and 3.0–3.8 mm ventral to bregma. Injections for these experiments were 100 nL per site. For schemes with mDlx‐ChR2 and rg‐mCherry injected into the same region, virus was combined in a 1:1 ratio. When using the mDlx‐ChR2 AAV, virus was allowed to incubate for at least 6 weeks before proceeding with experiments.

For experiments examining the firing patterns of SuM cells projecting to the medial septum (SuM→MS) AAVrg‐hSyn‐mCherry (rg‐mCherry) (Addgene viral prep # 114472‐AAVrg, generously donated by Karl Deisseroth, titre 1.9×10^12^ vg/mL) was injected at 0.75 mm anterior, 0.05 mm left and 4.1 mm ventral to bregma (*n* = 4 Bl6 mice, 2 females, 2 males). Slice electrophysiology targeting PV^+^ cells or NOS^+^ cells (*n* = 6 NOS‐Cre mice, 5 females, 1 male) was accomplished by injecting 200 nL of AAV8‐CAG‐FLEX‐tdTOMATO (UNC Viral Vector Core lot number AV4912C and AV4912B, titre 1.9×10^12^ vg/mL) into the SuM (15° angle tilted back towards posterior brain; 3.75 mm posterior, 0.5 mm left of bregma, 5 mm down the angled axis). Virus was allowed to incubate for 3 weeks before the experiments were performed.

We attempted to capture Hi→SuM neurons via retrograde AAV injected into the SuM; however all retrograde viruses tested failed to capture Hi→SuM projections, possibly due to this long‐range inhibitory population being incompatible with the retrograde serotype (Tervo et al., 2016). The tested viruses were as follows: AAVrg‐Ef1a‐mCherry‐IRES‐Cre (Addgene viral prep # 55632‐AAVrg, generously donated by Karl Deisseroth (Fenno et al., 2014), titre 1.3×10^13^ vg/mL; tested in a Cre‐reporter mouse line); AAVrg‐hSyn‐EGFP (Addgene viral prep # 50465‐AAVrg, generously donated by Bryan Roth, titre 1.0×10^13^ vg/mL); AAVrg‐hSyn‐mCherry (Addgene viral prep # 114472‐AAVrg, generously donated by Karl Deisseroth, titre 1.9×10^12^ vg/mL); AAVrg‐mDlx‐GFP‐Fishell‐1 (Addgene viral prep # 83900‐AAVrg, generously donated by Gordon Fishell (Dimidschstein et al., 2016), titre 1.7×10^13^ vg/mL); AAVrg‐mDlx‐NLS‐Ruby2 (Addgene viral prep # 99130‐AAVrg, generously donated by Viviana Gradinaru (Chan et al., 2017), titre 1.2×10^13^ vg/mL); AAVrg‐mDlx‐ChR2‐mCherry‐Fishell‐3 (Addgene viral prep # 83898‐AAVrg, generously donated by Gordon Fishell (Dimidschstein et al., 2016), titre 1.5×10^13^ vg/mL); and AAVrg‐FLEX‐tdTomato, injected into GAD‐Cre mice (Addgene viral prep # 28306‐AAVrg, CAG promoter, generously donated by Edward Boyden, titre 2.6×10^13^ vg/mL).

#### Combined fluorogold and virus injection

NOS‐Cre mice were injected with a Cre‐dependent AAV across the hippocampus and fluorogold into the SuM to assess the proportion of Hi→SuM cells which are also NOS^+^ (*n* = 3 mice). Mice were injected with AAVDJ‐mDlx‐DIO‐NLS‐mScarlet‐3xNLS (Addgene plasmid p164‐pAAV‐mDlx‐FLEX‐mScarlet, # 204689, generously donated by Lynette Lim (Fisher et al., 2024) and adapted and packaged by the UMN VVCC, 1.1×10^12^ vg/mL) as follows: 200 nL at 1.8 mm posterior, 1.4 mm left and 1.3 mm ventral to bregma; 200 nL at 2.0 mm posterior, 1.6 mm left and 2.0 mm ventral to bregma; 300 nL at 2.6 mm posterior and 2.8 mm left of bregma, in 100 nL increments at 4.1, 3.2 and 2.2 mm ventral to bregma; 300 nL at 2.9 mm posterior and 2.5 mm left of bregma, in 100 nL increments at 4.7, 3.0 and 1.75 mm ventral to bregma; 300 nL at 3.2 mm posterior and 3.45 mm left of bregma, in 100 nL increments at 4.2, 3.4 and 1.6 mm ventral to bregma; and 200 nL at 3.4 mm posterior and 2.5 mm left of bregma, in 100 nL increments at 4.2 and 2.9 mm ventral to bregma. Approximately 2 weeks later fluorogold was injected into the SuM as stated above in Fluorogold injections section. One week later the tissue was harvested, and somatostatin immunohistochemistry was performed as described below.

### Tissue processing, immunohistochemistry and cell counting

Whole‐brain tissue for quantification was collected and fixed in 4% PFA for 1–3 days at 4°C before a vibratome was used (Leica VT1000S) to section the tissue into 50 µm‐thick coronal sections.

#### Counting hippocampal cells projecting to the SuM

In tissue with FG labelling, immunohistochemistry (IHC) was performed to examine immunolabeling of SST (*n* = 5 mice), M2R (*n* = 4 mice), VIP (*n* = 3 mice), PV (*n* = 3 mice) and coexpression of SST (assessed using IHC) and NOS (assessed via viral injection in NOS‐Cre mice as above, *n* = 3 mice) in FG^+^ hippocampal neurons. Every fourth section was selected for quantification (*n* = 14 sections per mouse). The tissue was washed in 0.1M phosphate‐buffered solution (PB) and then in tris‐buffered saline (TBS, 0.9% NaCl) prior to incubation in one of the following antibodies: rat anti‐somatostatin (SST), Millipore Sigma MAB354, incubated in 1:250 dilution for 96 h; rabbit anti‐muscarinic cholinergic receptor 2 (M2R), EMD Millipore AB5166, incubated in 1:1000 dilution overnight; rabbit anti‐vasoactive intestinal peptide (VIP), Immunostar 20077, AB_572270, incubated in 1:500 dilution overnight; or rabbit anti‐parvalbumin, Swant PV27, incubated in 1:1000 dilution overnight. For SST, M2R, and VIP immunohistochemistry the tissue was incubated in a blocking buffer for 1 h prior to incubation in primary antibody (10% normal goat serum in TBS‐Triton 0.5% for M2R and VIP immunohistochemistry or 5% normal donkey serum in TBS‐Triton 0.1% for SST immunohistochemistry). For PV immunohistochemistry the tissue was incubated in 100% methanol for 3 h prior to standard washes and primary antibody incubation. After primary incubation the tissue was washed in TBS and incubated for 2 h at room temperature in one of the following Alexa fluor 594‐conjugated antibodies: donkey anti‐rat‐594, Jackson Immuno Research 112‐585‐167, 1:500 dilution; goat anti‐rabbit‐594, Jackson Immuno Research 111‐585‐003, 1:500 dilution. In tissue examining coexpression of SST and NOS an Alexa fluor 488‐conjugated antibody was used: donkey anti‐rat 488, Jackson Immuno Research 712‐546‐153, 1:500 dilution. All antibodies were diluted in TBS with Triton concentration ranging from 0.025% to 0.5%. The tissue was then washed in 0.1M PB and mounted on glass slides using Vectashield mounting media (H‐1000, Vector Laboratories, Inc).

The tissue was then visualized using a Leica DM2500 microscope, and FG^+^ cells were identified and checked for concurrent immunolabelling (i.e. 594‐positive cells representing SST^+^, PV^+^, VIP^+^ or M2R^+^ cells). In tissue examining overlap of SST IHC and viral NOS labelling, only the hippocampal tissue with adequate viral expression was quantified, excluding regions of the tissue where virus did not spread. Cells were mapped to the Franklin and Paxinos Mouse Brain Atlas (Franklin & Paxinos, 2007) and localized to hippocampal subregions (CA1, CA2, CA3, or DG) and their layers using baseline autofluorescence to identify anatomical region and AP location. Where data are plotted against the AP location (Fig. 1*C*), each point represents count of FG^+^ cells in two sections of the hippocampus, and AP location is the average AP distance from bregma of the two sections.

#### Terminal quantification

Sectioned tissue was mounted using Vectashield with DAPI (H‐1200, Vector Laboratories, Inc). A section of anterior SuM (2.8 mm posterior to bregma) and posterior SuM (3.0 mm posterior to bregma) were selected for quantification in each mouse, with the AP location determined by viewing DAPI fluorescence. For each section five ROIs distributed across ipsilateral, medial, and contralateral SuM were selected and imaged (*n* = 10 ROIs/mouse). The experimenter was blinded to fibre location while selecting regions to image and instead used co‐ordinates relative to the principal mammillary tract to select regions. Image stacks were collected using a Nikon A1Rsi microscope with a 60× oil immersion objective (MRD01605) at a 1024×1024 resolution and 0.1 µm pixel size. EGFP^+^ puncta were quantified in a 20 µm‐thick stack (step size 0.1 µm) using a custom pipeline generated in Nikon elements. This pipeline used Nikon Elements processes to denoise the image before setting a custom pixel threshold and minimum puncta size (0.10 µm diameter) to isolate EGFP^+^ puncta. Pixel thresholds selected varied between mice but were consistent across ROIs within a mouse. For data comparing puncta numbers between regions (e.g. ipsilateral *vs*. medial *vs*. contralateral, anterior *vs*. posterior) puncta from NOS‐Cre tissue were manually quantified in addition to the automated pipeline. This was possible due to the relatively low total number of puncta in NOS‐Cre tissue and gave more precision for comparing between SuM regions within NOS‐Cre mice. For a subset of SST‐Cre and NOS‐Cre mice, the number of labeled hippocampal soma were counted in hippocampal tissue from a 1‐in‐4 series across the anterior‐posterior axis of the hippocampus.

Data were analysed by applying a repeated‐measures, mixed‐effects ANOVA with the following mixed effects: between‐group effects of sex, virus, mouse line, and injection location, as well as the interaction between mouse line and injection location; and within‐subjects effects comparing AP SuM ROIs and ipsilateral‐medial‐contralateral SuM ROIs (Table A1). Mice were injected with one of two viruses (phSyn1‐SypEGFP *vs*. mDlx‐SypEGFP); we found no significant difference between viruses (main effect, *F*_(1,10)_ = 0.0001, *P* = 0.99), and the data were subsequently pooled. Between‐subjects effects are reported as *F*_(dfBS, dfBS(Error))_, where dfBS is the degrees of freedom for between‐subjects effects, and dfBS(Error) is the degrees of freedom for the error. Similarly within‐subjects effects are reported as *F_(_*_dfWS, dfWS(Error))_.

All statistical tests, including repeated‐measures ANOVA and *post hoc* testing with Tukey‐Kramer tests, were performed using MATLAB R2024b.

### Slice electrophysiology recordings

Mice used for slice electrophysiology were deeply anaesthetized with 5% isoflurane prior to harvesting tissue. The brain was then sectioned into 250 µm‐thick coronal sections (either flat coronal or angled, cut running from approximately 0.5 mm posterior of bregma at dorsal surface to 1 mm anterior of bregma at ventral surface) while in ice‐cold sucrose using a Leica VT1200 S vibratome. After sectioning, tissue was incubated for 30 min at 34°C in a solution with a 1:1 ratio of sucrose to ACSF. It was then allowed to rest at room temperature for at least 1 h before patching. The sucrose solution contained the following (in mM): 85 NaCl, 75 sucrose, 2.5 KCl, 25 glucose, 1.25 NaH_2_PO4, 4 MgCl_2_, 0.5 CaCl_2_ and 24 NaHCO_3_. The ACSF solution contained (in mM): 2.5 KCl, 10 glucose, 126 NaCl, 1.25 NaH_2_PO_4_, 2 MgCl_2_, 2 CaCl_2_ and 26 NaHCO_3_ (Krook‐Magnuson et al., 2011).

All recordings were done in ACSF at 36°C. Fluorescent SuM cells ipsilateral to the site of viral injections were identified for patching using an upright microscope (Nikon Eclipse FN1) with a xenon light source (Lambda DG‐4Plus, Sutter Instrument Company (Novato, CA, USA), model PE300BFA) (Krook‐Magnuson et al., 2011). Where relevant, non‐fluorescent cells in the same field of view as fluorescent cells were patched.

Recordings were performed using pipettes (3‐4 MΩ) filled with a high‐chloride, caesium‐free internal solution that allowed for recording both GABA_A_ and GABA_B_ currents while holding the cell at −60 mV. The intracellular solution contained the following (in mM): 90 potassium gluconate, 43.5 KCl, 1.8 NaCl, 1.7 MgCl_2_, 0.05 EGTA, 10 HEPES, 2 Mg‐ATP, 0.4 Na_2_‐GTP, 10 phosphocreatine and 8 biocytin, pH 7.29, 290 mOsm (Krook‐Magnuson et al., 2011).

All recordings were in the whole‐cell patch configuration. Resting membrane potential was recorded for each cell prior to any experimental manipulations. Firing properties of cells were identified by injecting a range of current steps, starting at −150 pA and increasing in steps of 25 or 5 pA (500 ms step duration). Where possible these recordings were done both at the cell's resting potential and then with baseline current injected to hold the cell around −60 mV. For optogenetic experiments cells were voltage clamped at −60 mV while blue light was flashed in a paired‐pulse pattern (5 ms light pulses given with a 20 ms interpulse interval, 10 s intersweep interval). Blue light was delivered through the microscope lens and controlled using the Lambda DG‐4Plus (Sutter Instrument Company, model PE300BFA). Series resistance was monitored across recordings, and data were excluded if series resistance was above 30 MΩ.

In a subset of experiments, recorded light‐evoked responses were followed by bath application of the GABA_A_ receptor antagonist gabazine (5 µM, Sigma‐Aldrich CAS Number: 104104‐50‐9). All responses were eliminated after application of gabazine. When gabazine was used, tissue was immediately fixed without further patching. There were no observed slow postsynaptic responses characteristic of GABA_B_ currents in response to light stimulation.

After recordings, tissue was fixed in 4% PFA for 1–3 days at 4°C. Patched cells were recovered and visualized with a 2 h incubation in streptavidin‐405 (Jackson ImmunoResearch, 016‐470‐084, 1:500 dilution).

#### Electrophysiology analysis

Data were collected using pCLAMP Clampex software (version 10.7, Axon Instruments) and then reviewed in Clampfit (version 11.4, Axon Instruments) or exported to MATLAB. Light‐evoked PSCs were identified visually as evoked responses with consistent onset latency after blue light pulses, repeatedly evoked on at least five separate sweeps. The onset latency and amplitude were visually identified in averaged traces and measured using Clampfit software. All amplitude measures occurred while holding the cell at −60 mV. Paired pulse ratios were calculated based on the amplitude measures, including failed release, using the formula (amplitude_pulse2 − amplitude_pulse1)/(amplitude_pulse2 + amplitude_pulse1). Burst‐wave firing SuM cells were identified by visualizing the first positive current step evoking two or more action potentials; burst‐wave cells displayed two or more action potentials with a short interspike interval followed by an extended afterhyperpolarization.

All statistical tests were performed using MATLAB R2024b. All averaged data are represented as mean ± SEM. All analyses comparing light‐evoked response properties (amplitude and onset latency of PSCs) or cell properties (resting membrane potential) between cell types were compared using a two‐tailed MWU. Comparisons of the proportion of cells with light‐evoked responses were made using either a Fisher exact test (when count of light‐evoked PSCs was < 10) or χ^2^ test (when count of light‐evoked PSCs was 10 or more). The evoked firing rate during progressive steps of injected current was compared between cell populations with a repeated‐measures ANOVA.

## Additional information

## Competing interests

None declared.

## Author contributions

L.G. and E.K.M. conceived the work and designed the experiments. L.G., E.H. and O.S. contributed to data collection and analysis. E.M.F.d.V. designed and created the AAV used for optogenetic experiments. L.G. and E.K.M. drafted and revised this article. All authors gave feedback on the final manuscript.

## Funding

This work and the Krook‐Magnuson lab was supported by National Institutes of Health grants R01NS104071 (to Esther Krook‐Magnuson), R01NS139469 (to Esther Krook‐Magnuson) and F31NS127417 (to Lauren Glassburn), as well as MnDRIVE (Minnesota's Discovery, Research, and Innovation Economy) initiative (to Esther Krook‐Magnuson) and Neuromodulation Fellowship (to Lauren Glassburn) and a McKnight Land‐Grant Professorship and Presidential Fellow (to Esther Krook‐Magnuson).

## Generative AI statement

No generative AI tools were used in the preparation of this manuscript.

## Supporting information


Peer Review History

## Data Availability

All relevant data and analysis are available from the corresponding author upon reasonable request. Imaging files used for quantifying SypEGFP^+^ synapses and slice electrophysiology data from recorded SuM cells are publicly available on DANDI, ID: 001510.

## Appendix

**Table A1:** Hippocampal innervation of the supramammillary area (SuM) varies between inhibitory cell types, hippocampal subregions and SuM target subregions

Repeated‐measures analysis of variance – effects on number of SypEGFP+ puncta in SuM							
Between‐subjects effects							
Main effect**Interaction*	Number of mice with experimental scheme (*n* = 16 mice total)	SumSq	DF	MeanSq	*F*	*P‐*value	Significance
Intercept		1.46E+05	1	1.46E+05	139.19	< 0.001	***
Hippocampal cell type	*n* = 8 SST‐Cre mice injected, 8 NOS‐Cre mice injected	80,058	1	80,058	76.58	< 0.001	***
Hippocampal subregion	*n* = 8 dHi, 8 vHi injected mice	24,182	1	24,182	23.13	< 0.001	***
Sex	*n* = 8 female, 8 male mice	247.51	1	247.51	0.24	0.637	ns
Virus	*n* = 8 mDlx‐SypEGFP virus, 8 phSyn1(S)‐SypEGFP injected mice	0.16	1	0.16	< 0.001	0.990	ns
*Hippocampal cell type***hippocampal subregion*		32,291	1	32,291	30.89	< 0.001	***
Error		10,454	10	1045.4			

Within‐subjects effects							
Main effect**Interaction*	Number of ROIs	SumSq	DF	MeanSq	*F*	*P*‐value	Significance
Intercept(anterior *vs*. posterior SuM)	*n* = 5 anterior, 5 posterior ROIs/mouse	660.16	1	660.16	0.28	0.607	ns
Error(anterior *vs*. posterior SuM)		23,420	10	2342			
*Anterior vs. posterior***hippocampal cell type*		88.51	1	88.51	0.04	0.850	ns
*Anterior vs. posterior***sex*		644.01	1	644.01	0.27	0.611	ns
*Anterior vs. posterior***hippocampal subregion*		860.26	1	860.26	0.37	0.558	ns
*Anterior vs. posterior***Virus*		4090.5	1	4090.5	1.7466	0.216	ns
*Anterior vs. posterior***hippocampal cell type***hippocampal subregion*		425.76	1	425.76	0.18	0.679	ns
Intercept(lateral–medial SuM)	*n* = 4 ipsilateral, 2 medial, 4 contralateral ROIs/mouse	1.01E+05	2	50,508	92.32	< 0.001	***
Error(lateral–medial SuM)		10,942	20	547.11			
*Lateral–medial***hippocampal cell type*		63,800	2	31,900	58.31	< 0.001	***
*Lateral–medial***sex*		619.64	2	309.82	0.57	0.576	ns
*Lateral–medial***hippocampal subregion*		26,981	2	13,490	24.66	< 0.001	***
*Lateral–medial***virus*		3983	2	1991.5	3.64	0.0449	*
*Lateral–medial***cell type***hippocampal subregion*		29,692	2	14,846	27.14	< 0.001	***

*Notes*: Synaptophysin‐tagged EGFP (SypEGFP) was expressed in hippocampal SST^+^ or hippocampal NOS^+^ cells in either dorsal or ventral hippocampus (see Fig. 2). SypEGFP is naturally trafficked to synapses, allowing for quantification of innervation arising from the hippocampus. The number of SypEGFP+ synapses in the SuM was quantified for each mouse, with multiple regions of interest (ROI) spread across subregions of the SuM. A repeated‐measures ANOVA was performed to assess if hippocampal innervation of the SuM varied between experimental schemes and between subregions of the SuM, finding that SuM innervations vary between hippocampal cell types (SST^+^ *vs*. NOS^+^ cells), hippocampal subregions (dorsal *vs*. ventral hippocampal neurons) and SuM subregions (ipsilateral *vs*. medial *vs*. contralateral SuM). SumSq, sum of squares; DF, degrees of freedom; MeanSq, mean squares.

**P* < 0.05; ****P* < 0.001. ns, not significant.

**Table A2:** SST^+^ hippocampal neurons in ventral hippocampus densely innervate lateral supramammillary area (SuM)

SuM innervation arising from hippocampal subpopulations – number of puncta per SuM ROI, *post hoc* testing					
Group 1	Group 2	Mean ± SEM 1	Mean ± SEM 2	Tukey‐Kramer: *P*‐value	Significance
***SST^+^ cells, from ventral Hi***	NOS^+^ cells, from dorsal Hi	79.0 ± 5.7	9.7 ± 2.5	< 0.001	***
***SST^+^ cells, from ventral Hi***	NOS^+^ cells, from ventral Hi	79.0 ± 5.7	5.9 ± 1.6	< 0.001	***
***SST^+^ cells, from ventral Hi***	SST^+^ cells, from dorsal Hi	79.0 ± 5.7	26.0 ± 7.1	< 0.001	***
SST^+^ cells, from dorsal Hi	NOS^+^ cells, from dorsal Hi	26.0 ± 7.1	9.7 ± 2.5	0.121	ns
***SST^+^ cells, from dorsal Hi***	NOS^+^ cells, from ventral Hi	26.0 ± 7.1	5.9 ± 1.6	0.0460	*
NOS^+^ cells, from ventral Hi	NOS^+^ cells, from dorsal Hi	5.9 ± 1.6	9.7 ± 2.5	0.938	ns
**Innervation of anterior *vs*. posterior SuM – total puncta count, *post hoc* testing**					
SST^+^ hippocampal cells, all					
Anterior	Posterior	248.8 ± 55.7	276.5 ± 73.3	0.680	ns
NOS+ hippocampal cells, all					
Anterior	Posterior	128.6 ± 20.4	166.9 ± 34.9	0.571	ns
					
**Innervation of lateral *vs*. medial SuM – number of puncta per ROI, *post hoc* testing**					
SST+ hippocampal cells, from dorsal hippocampus					
Ipsilateral	Contralateral	38.8 ± 10.7	25.3 ± 9.0	0.574	ns
***Ipsilateral***	Medial	38.8 ± 10.7	2.1 ± 1.4	0.0149	*
Medial	***Contralateral***	2.1 ± 1.4	25.3 ± 9.0	0.0438	*
SST+ hippocampal cells, from ventral hippocampus					
***Ipsilateral***	Contralateral	146.6 ± 12.2	41.2 ± 8.7	< 0.001	***
***Ipsilateral***	Medial	146.6 ± 12.2	19.6 ± 3.3	< 0.001	***
Medial	Contralateral	19.6 ± 3.3	41.2 ± 8.7	0.0606	ns
NOS+ hippocampal cells, from dorsal hippocampus					
Ipsilateral	Contralateral	47.7 ± 14.3	32.6 ± 6.9	0.505	ns
***Ipsilateral***	Medial	47.7 ± 14.3	4.9 ± 3.3	0.00543	**
Medial	***Contralateral***	4.9 ± 3.3	32.6 ± 6.9	0.0164	*
NOS+ hippocampal cells, from ventral hippocampus					
Ipsilateral	Contralateral	40.1 ± 8.6	15.9 ± 1.9	0.199	ns
Ipsilateral	Medial	40.1 ± 8.6	18.0 ± 8.5	0.151	ns
Medial	Contralateral	18.0 ± 8.5	15.9 ± 1.9	0.968	ns

*Notes*: Hippocampal innervation of SuM subregions was quantified by counting virally labelled synapses in the SuM, originating from various subpopulations of hippocampal neurons (see Fig. 2 for experimental schemes). *Post hoc* Tukey‐Kramer tests were used to compare innervation of the SuM from specific hippocampal subpopulations and also to compare innervation within SuM subregions (see Table A1 for repeated‐measures ANOVA results). Where relevant the tested group with significantly greater innervation is bolded and italicized.

* *P* < 0.05; ***P* < 0.01; ****P* < 0.001. ns, not significant.
