# Supplementary material for: Hippocampal long‐range GABAergic neurons target hippocampal‐projecting cells of the supramammillary area
Source: J Physiol. 2026 Sep 11;604(18):7924–56. doi: 10.1113/JP291809 (PMC13577689; doi:10.1113/JP291809)
Supplement: Supplementary file 1 [file TJP-604-7924-s001.pdf]

The Journal of Physiology JP

Hippocampal long-range GABAergic neurons target hippocampal–projecting cells of the supramammillary area

Lauren Glassburn, Edmund Havener, Olivia Stangler, Ezequiel Marron Fernandez de Velasco, and Esther Krook-Magnuson
DOI: 10.1113/JP291809

Corresponding author(s): Lauren Glassburn (glass233@umn.edu)

The following individual(s) involved in review of this submission have agreed to reveal their identity: Justin James Botterill (Referee #1)

Review Timeline:

Submission Date:	26-May-2026
Editorial Decision:	02-Jul-2026
Revision Received:	06-Aug-2026
Accepted:	20-Aug-2026

Senior Editor: Jing-Ning Zhu

Reviewing Editor: Ming Yi

Transaction Report:

(Note: With the exception of the correction of typographical or spelling errors that could be a source of ambiguity, letters and reports are not edited. Depending on transfer agreements, referee reports obtained elsewhere may or may not be included in this compilation. Referee reports are anonymous unless the Referee chooses to sign their reports.)

Re: JP-RP-2026-291809 "Hippocampal long-range GABAergic neurons target hippocampal-projecting cells of the supramammillary area" by Lauren Glassburn, Edmund Havener, Olivia Stangler, Ezequiel Marron Fernandez de Velasco, and Esther Krook-Magnuson

Dear Dr Glassburn,

Thank you for submitting your manuscript to The Journal of Physiology. It has been assessed by a Reviewing Editor and by 2 expert referees and we are pleased to tell you that it is potentially acceptable for publication following satisfactory major revision.

Please advise your co-authors of this decision as soon as possible.

The referee reports are copied at the end of this email.

Please address all the points raised and incorporate all requested revisions or explain in your Response to Referees why a change has not been made. We hope you will find the comments helpful and that you will be able to return your revised manuscript within 2 months. If your article is NOT for a Special Issue, you may have 9 months to revise. If you require an extension, please contact journal staff: jp@physoc.org. Please note that this letter does not constitute a guarantee for acceptance of your revised manuscript.

Your revised manuscript should be submitted online using the link in your Author Tasks: Link Not Available. This link is accessible via your account as Corresponding Author; it is not available to your co-authors. If this presents a problem, please contact journal staff (jp@physoc.org). Image files from the previous version are retained on the system. Please ensure you replace or remove any files that are being revised.

If you do not wish to submit a revised version of your manuscript, you must inform our journal staff (jp@physoc.org) or reply to this email to request withdrawal. Please note that a manuscript must be formally withdrawn from the peer review process at one journal before it may be submitted to another journal.

TRANSPARENT PEER REVIEW POLICY: To improve the transparency of its peer review process, The Journal of Physiology publishes online as supporting information the peer review history of all articles accepted for publication. Readers will have access to decision letters, including Editors' comments and referee reports, for each version of the manuscript, as well as any author responses to peer review comments. Referees can decide whether or not they wish to be named on the peer review history document.

ABSTRACT FIGURES: Authors are expected to use The Journal's premium BioRender account to create/redraw their Abstract Figures. Information on how to access this account is here: https://physoc.onlinelibrary.wiley.com/journal/14697793/biorender-access.

This will enable Authors to create and download high-resolution figures. If authors have used the free BioRender service, they can use the instructions provided in the link above to download a high-resolution version suitable for publication.

The link provided should only be used for the purposes of this submission. Authors will be charged for figures created on this account if they are not related to this manuscript submission.

LANGUAGE EDITING AND SUPPORT FOR PUBLICATION: If you would like help with English language editing, or other article preparation support, Wiley Editing Services offers expert help, including English Language Editing, as well as translation, manuscript formatting, and figure formatting at www.wileyauthors.com/eeo/preparation. You can also find resources for Preparing Your Article for general guidance about writing and preparing your manuscript at www.wileyauthors.com/eeo/prepresources.

RIGOUR AND REPRODUCIBILITY: We recommend authors consult The Journal's Rigour and Reproducibility requirements to ensure that their manuscript is suitable for publication: https://physoc.onlinelibrary.wiley.com/pb-assets/hub-assets/physoc/documents/TJP-Rigour-and-Reproducibility-Requirements.pdf

REVISION CHECKLIST:

Check that your Methods section conforms to journal policy: https://jp.msubmit.net/cgi-bin/main.plex?form_type=display_requirements#methods.

Check that data presented conforms to the statistics policy: https://jp.msubmit.net/cgi-bin/main.plex?form_type=display_requirements#statistics.

Upload a full Response to Referees file. To create your 'Response to Referees': copy all the reports, including any comments from the Senior and Reviewing Editors, into a Microsoft Word, or similar, file and respond to each point, using font or background colour to distinguish comments and responses and upload as the required file type.

Please upload two versions of your manuscript text: one with all relevant changes highlighted and one clean version with no changes tracked. The manuscript file should include all tables and figure legends, but each figure/graph should be uploaded as separate, high-resolution files.

You may also upload:

- 'Potential Cover Art' for consideration as the issue's cover image
- Appropriate Supporting Information (Video, audio or data set: see https://jp.msubmit.net/cgi-bin/main.plex?form_type=display_requirements#supp).

We look forward to receiving your revised submission.

If you have any queries, please reply to this email and we will be pleased to advise.

Yours sincerely,

Jing-Ning Zhu
Senior Editor
The Journal of Physiology

REQUIRED ITEMS

- Author photo and profile. First or joint first authors are asked to provide a short biography (no more than 100 words for one author or 150 words in total for joint first authors) and a portrait photograph. These should be uploaded and clearly labelled together in a Word document with the revised version of the manuscript. See Information for Authors for further details.

- You must start the Methods section with a paragraph headed Ethical approval (https://jp.msubmit.net/cgi-bin/main.plex?form_type=display_requirements#methods).

Research must comply with The Journal's policies regarding animal experiments (https://physoc.onlinelibrary.wiley.com/hub/animal-experiments) and adherence to these policies must be stated in the manuscript.

Authors should confirm in their Methods section that their experiments were carried out according to the guidelines laid down by their institution's animal welfare committee, including an ethics approval reference number. The Methods section must contain a statement about access to food, water and housing, details of the anaesthetic regime: anaesthetic used, dose and route of administration, and method of killing the experimental animals.

- The reference list must be in alphabetical order, rather than numbered, to comply with our Journal format.

- Your manuscript must include a complete Additional Information section, including competing interests; funding; author contributions and acknowledgements.

- The Journal of Physiology funds authors of provisionally accepted papers to use the premium BioRender site to create high resolution schematic figures. Follow this link and enter your details and the manuscript number to create and download figures. Upload these as the figure files for your revised submission. If you choose not to take up this offer, we require figures to be of similar quality and resolution. If you are opting out of this service to authors, state this in the Comments section on the Detailed Information page of the submission form. The link provided should only be used for the purposes of this submission. Authors will be charged for figures created on this premium BioRender account if they are not related to this manuscript submission.

- Please upload separate high-quality figure files via the submission form.

- Please ensure that any tables are editable and in Word format, and wherever possible, embedded in the article file itself.

- Please ensure that the Article File you upload is a Word file.

- Your paper contains supplementary tables. Please include these at the bottom of the manuscript as part of an Appendix. Tables should be numbered as Table A1, etc. - and each should be cited in the text.

- Papers must comply with the Statistics Policy: https://jp.msubmit.net/cgi-bin/main.plex?form_type=display_requirements#statistics.

In summary:

- If n {less than or equal to} 30, all data points must be plotted in the figure in a way that reveals their range and distribution. A bar graph with data points overlaid, a box and whisker plot or a violin plot (preferably with data points included) are acceptable formats.

- If n > 30, then the entire raw dataset must be made available either as supporting information, or hosted on a not-for-profit repository, e.g. FigShare, with access details provided in the manuscript.

- 'n' clearly defined (e.g. x cells from y slices in z animals) in the Methods. Authors should be mindful of pseudoreplication.

- All relevant 'n' values must be clearly stated in the main text, figures and tables.

- The most appropriate summary statistic (e.g. mean or median and standard deviation) must be used. Standard Error of the Mean (SEM) alone is not permitted.

- Exact p values must be stated. Authors must not use 'greater than' or 'less than'. Exact p values must be stated to three significant figures even when 'no statistical significance' is claimed.

- Please include an Abstract Figure file and an Abstract Figure legend. An appropriate figure legend, which should not exceed 150 words in length, should be included in the main manuscript file. The Abstract Figure is a piece of artwork designed to give readers an immediate understanding of the research and should summarise the main conclusions. If possible, the image should be easily 'readable' from left to right or top to bottom. It should show the physiological relevance of the manuscript so readers can assess the importance and content of its findings. Abstract Figures should not merely recapitulate other figures in the manuscript. Please try to keep the diagram as simple as possible and without superfluous information that may distract from the main conclusion(s). Abstract Figures must be provided by authors no later than the revised manuscript stage and should be uploaded as a separate file during online submission labelled as File Type 'Abstract Figure'. Please also ensure that you include the figure legend in the main article file. All Abstract Figures should be created using BioRender. Authors should use The Journal's premium BioRender account to export high-resolution images. Details on how to use and access the premium account are included as part of this email.

- Authors are required to fully disclose the use of any Generative AI (GAI) tools used in the preparation of their manuscript. This disclosure should also be included as a statement alongside the Additional Information and Data Availability statements towards the end of the article under the heading 'Generative AI Statement', and include the name(s), model and version of AI technology used, and exact details on the purpose for, and methods of, its use.

If no AI tool has been used, this should also be specifically stated within the manuscript.

EDITOR COMMENTS

Please also see 'Required Items' above.

Reviewing Editor: Comments to the Author (Required):

Based on the referees' comments, I would like to recommend a revision of the manuscript. Please carefully read the comments, make necessary revisions, and provide a point-to-point response letter along with the revised manuscript.

Senior Editor:

Comments to the Author:
Thank you for submitting your manuscript to The Journal of Physiology. Both reviewers and the reviewing editor found your work timely and important, providing a thorough investigation of a novel inhibitory hippocampal-supramammillary nucleus circuit. However, they have raised several points that require attention before we can proceed with publication. I look forward to your revision.

REFEREE COMMENTS

Referee #1:

Summary:

In this work, Glassburn and colleagues describe novel inhibitory hippocampal projections to the supramammillary nucleus of the hypothalamus (SuM). Considerable work to date has focused on SuM projections to the dentate gyrus and CA2 subfield of the hippocampus and their role in diverse behaviours and network oscillations. However, there is limited data on hippocampal projections to the SuM. The authors demonstrate proof of this novel hippocampal-SuM projection through a combination of well-validated approaches. First, the authors performed retrograde fluorogold labeling coupled with immunofluorescence staining to identify the neurochemical identity of hippocampal-SuM projecting cells. These experiments found that SST+ cells from a variety of hippocampal subfields project to the SuM and allowed the authors to rule out other major cell types (e.g., VIP+, PV+, M2R+, etc). Subsequent experiments utilized injections of Cre-dependent viral vectors in SST-Cre or NOS-Cre mice and confirmed that SST+ and NOS+ cells are the major hippocampal cell types that project to the SuM. Next, the authors used an optogenetic construct specific for inhibitory neurons (mDLX-ChR2), electrophysiological recordings, immunofluorescence staining of biocytin-filled patched cells, and different mouse Cre lines to show that inhibitory hippocampal projections to the SuM innervate neurochemically diverse SuM neurons (Tac1+, Tac1-, and PV+ cells). Additional viral labeling experiments demonstrate that the inhibitory hippocampal to SuM projections innervate SuM neurons that project back to the hippocampus. Slice electrophysiology combined with optogenetics further confirmed that these inhibitory hippocampal to SuM projections are sensitive to GABA A receptor antagonists such as Gabazine. Lastly, electrophysiological experiments showed that inhibitory hippocampal projections to the SuM preferentially target burst firing cells, which are implicated in physiologically-relevant processes, such as sharp wave ripples and theta-phase activity. Taken together, the authors provide a thorough investigation of this novel inhibitory hippocampal-SuM circuit.

Comments:

1. This is an excellent submission with minor issues. The anatomical and electrophysiological data are convincing and rigorous. My main criticism is that the anatomical and electrophysiological data are unable to demonstrate the relevance/function of this bi-directional circuit in vivo, but I also acknowledge that such experiments are well beyond the focus of the current submission.

2. The manuscript mentions that postoperative monitoring was done for 3 days after surgery, but no details are provided. If animals were given analgesics or drugs during the recovery period (e.g., metacam, ketoprofen, etc), this should be stated as per journal requirements checklist.

3. Figure 4, Panel B: The title for "Retrograde labeling" is partially cropped on the .PDF file. Please update the figure to ensure that 'Retrograde' is fully visible in the resubmitted figure.

4. Figure 4, Panel B: the mDLX-ChR2 panel text says "from ventral Hp" or "from dorsal Hp". However, "ventral Hi" and "dorsal Hi" have been used throughout the manuscript and in the figure legend. Please update the figure to use "Hi" instead of "Hp" to be consistent.

5. Some of the writing can be quite technical and written for an expert audience. The authors may want to consider some light edits to better contextualize the rationale for some of the more technical experiments (e.g., Figures 3-5).

Referee #2:

Glassburn et al., investigate the connectivity of long-range hippocampal GABAergic cells to understand whether they exhibit preferential targeting to any known classes of supramammillary (SuM) cells. It has been understood that there are sparse hippocampal projections to the SuM, but very little understood about the precise cell types or targeting logic. They use a combination of AAVs and tracing tools to label projections and confirm connectivity using whole-cell patch clamp and optogenetically evoked transients, which I find to be quite rigorous. The main conclusion is that there is heterogeneity in the GABAergic cell classes that target the SuM, among the known long-range classes (which this lab helped to define - namely LINCs), and that this input is selective for hippocampal projecting SuM neurons (its thought that not all SuM neurons target the hippocampus). Given the growing interest in SuM, I believe this work is timely and important and helps guide future functional experiments. I have a few small comments that should be addressed to strengthen the claims and sharpen the interpretations of the manuscript.

1. It is important to show images or injection-site maps for all mice used in fluorogold tracing, given how heterogenous and small the SuM is (also to rule out labelling in neighboring structures).

2. For Fig 2., it would be useful to understand how many cells were labelled in the hippocampus. This is important for the overall interpretation, since SST could provide stronger SuM innervation simply because there are more cells labelled.

3. Additionally, it would be useful to know whether the NOS+ and SST+ cells are mutually exclusive. In the discussion, it mentions how most NOS+ cells are negative for SST, and presumably the opposite is true, but that might depend on the density of labelling for each cell type (and perhaps by hippocampal region).

4. For Fig. 3, it would be useful to know if there was any preferential location of where in the SuM cells were patched, similar to Figure 4 (if possible). For example, the Tac1+ cell density is highest in the medial SuM where fibers are quite sparse and one may expect a lower probability of connection there. If cells in the medial SuM were not included, it could give the impression that Tac1+ are more strongly innervated than they truly are. Perhaps a discussion point.

5. You may be underpowered for this, but it would be interesting to know if the SuM cells that display rebound bursting are more or less likely to receive hippocampal input (perhaps even ignoring neurochemical markers).

6. Just like how it is noted that retroAAVs do not work for labelling long-range hippocampal GABAergic cells, the same is true for SuM cell types. The low density of labelling for SuM neurons and their axons back into the hippocampus seems to support this possibility (nowhere near the typical density of anterograde labelling).I believe it is crucial to acknowledge that nonfluorescent cells could still project to the hippocampus, and that the presence of labelling could reflect cell type-specific affinities for viral transduction (still very interesting).

7. Very minor - Fig 4, the top of the figure is cut off where it says "Retrograde"

END OF COMMENTS

We would like to thank the reviewers for their thoughtful reviews of the submitted manuscript. We have added the additional requested quantification, completed additional experiments, and updated figures and text in response to reviewer comments. A detailed response to reviewers' comments is below. Reviewer comments are in gray; responses are in blue. Please note that changes to the manuscript to address reviewer comments and to meet journal requirements (including providing SDs and p-values to three significant digits) are similarly displayed in blue in a version of the resubmitted manuscript. Line numbers in the response to reviewers below refer to line numbers provided in the track-changes version of the resubmitted manuscript.

REFEREE COMMENTS

Referee #1:

Summary: In this work, Glassburn and colleagues describe novel inhibitory hippocampal projections to the supramammillary nucleus of the hypothalamus (SuM). Considerable work to date has focused on SuM projections to the dentate gyrus and CA2 subfield of the hippocampus and their role in diverse behaviours and network oscillations. However, there is limited data on hippocampal projections to the SuM. The authors demonstrate proof of this novel hippocampal-SuM projection through a combination of well-validated approaches. First, the authors performed retrograde fluorogold labeling coupled with immunofluorescence staining to identify the neurochemical identity of hippocampal-SuM projecting cells. These experiments found that SST+ cells from a variety of hippocampal subfields project to the SuM and allowed the authors to rule out other major cell types (e.g., VIP+, PV+, M2R+, etc). Subsequent experiments utilized injections of Cre-dependent viral vectors in SST-Cre or NOS-Cre mice and confirmed that SST+ and NOS+ cells are the major hippocampal cell types that project to the SuM. Next, the authors used an optogenetic construct specific for inhibitory neurons (mDLX-ChR2), electrophysiological recordings, immunofluorescence staining of biocytin-filled patched cells, and different mouse Cre lines to show that inhibitory hippocampal projections to the SuM innervate neurochemically diverse SuM neurons (Tac1+, Tac1-, and PV+ cells). Additional viral labeling experiments demonstrate that the inhibitory hippocampal to SuM projections innervate SuM neurons that project back to the hippocampus. Slice electrophysiology combined with optogenetics further confirmed that these inhibitory hippocampal to SuM projections are sensitive to GABA A receptor antagonists such as Gabazine. Lastly, electrophysiological experiments showed that inhibitory hippocampal projections to the SuM preferentially target burst firing cells, which are implicated in physiologically-relevant processes, such as sharp wave ripples and theta-phase activity. Taken together, the authors provide a thorough investigation of this novel inhibitory hippocampal-SuM circuit.

Comments:

1. This is an excellent submission with minor issues. The anatomical and electrophysiological data are convincing and rigorous. My main criticism is that the anatomical and electrophysiological data are unable to demonstrate the relevance/function of this bi-directional

circuit in vivo, but I also acknowledge that such experiments are well beyond the focus of the current submission.

We agree that understanding the *in vivo* impact of this circuit would be very interesting, and that it is unfortunately beyond the scope of the current manuscript.

2. The manuscript mentions that postoperative monitoring was done for 3 days after surgery, but no details are provided. If animals were given analgesics or drugs during the recovery period (e.g., metacam, ketoprofen, etc), this should be stated as per journal requirements checklist.

Done. Information on post-operative care has been added (lines 658-662).

3. Figure 4, Panel B: The title for "Retrograde labeling" is partially cropped on the .PDF file. Please update the figure to ensure that 'Retrograde' is fully visible in the resubmitted figure.

Done. This has been corrected.

4. Figure 4, Panel B: the mDLX-ChR2 panel text says "from ventral Hp" or "from dorsal Hp". However, "ventral Hi" and "dorsal Hi" have been used throughout the manuscript and in the figure legend. Please update the figure to use "Hi" instead of "Hp" to be consistent.

Done. "Hi" is now used consistently throughout the manuscript.

5. Some of the writing can be quite technical and written for an expert audience. The authors may want to consider some light edits to better contextualize the rationale for some of the more technical experiments (e.g., Figures 3-5).

Done. We have edited writing to hopefully be accessible to a more general audience; example edits include lines 254-257 and 267-270.

<u>Referee #2:</u>
Glassburn et al., investigate the connectivity of long-range hippocampal GABAergic cells to understand whether they exhibit preferential targeting to any known classes of supramammillary (SuM) cells. It has been understood that there are sparse hippocampal projections to the SuM, but very little understood about the precise cell types or targeting logic. They use a combination of AAVs and tracing tools to label projections and confirm connectivity using whole-cell patch clamp and optogenetically evoked transients, which I find to be quite rigorous. The main conclusion is that there is heterogeneity in the GABAergic cell classes that target the SuM, among the known long-range classes (which this lab helped to define - namely LINCs), and that this input is selective for hippocampal projecting SuM neurons (its thought that not all SuM

neurons target the hippocampus). Given the growing interest in SuM, I believe this work is timely and important and helps guide future functional experiments. I have a few small comments that should be addressed to strengthen the claims and sharpen the interpretations of the manuscript.

1. It is important to show images or injection-site maps for all mice used in fluorogold tracing, given how heterogenous and small the SuM is (also to rule out labelling in neighboring structures).

Done. An injection site map has been added to Figure 1A.

2. For Fig 2., it would be useful to understand how many cells were labelled in the hippocampus. This is important for the overall interpretation, since SST could provide stronger SuM innervation simply because there are more cells labelled.

Done. A supplemental figure has been added with a sample count of labeled cells in a 1 in 4 series of the hippocampus from one mouse in each scheme (dorsal SST, dorsal NOS, ventral SST, ventral NOS) (Supp. Figure A1).

3. Additionally, it would be useful to know whether the NOS+ and SST+ cells are mutually exclusive. In the discussion, it mentions how most NOS+ cells are negative for SST, and presumably the opposite is true, but that might depend on the density of labelling for each cell type (and perhaps by hippocampal region).

Done. To address this point, we have completed additional experiments with simultaneous fluorogold retrograde tracing, viral labeling of NOS+ cells (via a cre-dependent AAV in NOS-Cre mice), and SST immunohistochemistry. This data suggests that there are populations of Hi→SuM cells which do express both NOS and SST, as well as populations that express only NOS or only SST. Data is discussed in lines 213-233 and presented in Supplemental Figure A1.

4. For Fig. 3, it would be useful to know if there was any preferential location of where in the SuM cells were patched, similar to Figure 4 (if possible). For example, the Tac1+ cell density is highest in the medial SuM where fibers are quite sparse and one may expect a lower probability of connection there. If cells in the medial SuM were not included, it could give the impression that Tac1+ are more strongly innervated than they truly are. Perhaps a discussion point.

Done. To address this relevant point, we have added a panel to Figure 3 with a map of patched Tac1+, Tac1-, and PV+ cells. We've also indicated in the text that our patch electrophysiology was focused on the lateral SuM, where we observe more Hi→SuM

fibers, and adjusted the text to emphasize that the proportion of connectivity applies to the regions we patched (e.g. lines 261-262, 286-287, 534-535).

5. You may be underpowered for this, but it would be interesting to know if the SuM cells that display rebound bursting are more or less likely to receive hippocampal input (perhaps even ignoring neurochemical markers).

Done. We have further analyzed if hippocampal inhibition preferentially targets rebound spiking cells, both in experiments where mDlx-ChR2 was expressed in inhibitory neurons across hippocampal subregions (patching Tac1 +/- cells; additional analysis now provided in supplemental Figure A2, panel D) and in experiments where mDlx-ChR2 was expressed in either dorsal or ventral hippocampus (patching SuM→Hi and unlabeled cells; additional analysis now provided in Figure 5F). In each of these experiments, there was no observed difference in innervation of rebound spiking vs. non-rebound spiking cells (lines 447-459).

6. Just like how it is noted that retroAAVs do not work for labelling long-range hippocampal GABAergic cells, the same is true for SuM cell types. The low density of labelling for SuM neurons and their axons back into the hippocampus seems to support this possibility (nowhere near the typical density of anterograde labelling).I believe it is crucial to acknowledge that nonfluorescent cells could still project to the hippocampus, and that the presence of labelling could reflect cell type-specific affinities for viral transduction (still very interesting).

Done. The fact that "nonfluorescent SuM cells" may include unlabeled SuM→Hi cells has been included in the discussion (lines 573-581) and emphasized in the main results text (lines 359).

7. Very minor - Fig 4, the top of the figure is cut off where it says "Retrograde"

Done. This has been corrected.

Dear Ms Glassburn,

Re: JP-RP-2026-291809R1 "Hippocampal long-range GABAergic neurons target hippocampal-projecting cells of the supramammillary area" by Lauren Glassburn, Edmund Havener, Olivia Stangler, Ezequiel Marron Fernandez de Velasco, and Esther Krook-Magnuson

We are pleased to tell you that your paper has been accepted for publication in The Journal of Physiology.

The last Word (or similar) version of the manuscript provided will be used by the Production Editor to prepare your proof. When this is ready you will receive an email containing a link to Wiley's Online Proofing System. The proof should be thoroughly checked and corrected as promptly as possible.

Authors should note that it is too late at this point to offer corrections prior to proofreading. Major corrections at proof stage, such as changes to figures, will be referred to the Editors for approval before they can be incorporated. Only minor changes, such as to style and consistency, should be made at proof stage. Changes that need to be made after proof stage will usually require a formal correction notice.

All queries at proof stage should be sent to: TJP@wiley.com.

Following copyediting and typesetting the accepted version will be published online.

Yours sincerely,

Jing-Ning Zhu
Senior Editor
The Journal of Physiology

IMPORTANT POINTS TO NOTE FOLLOWING ACCEPTANCE OF YOUR PAPER:

• IMPORTANT NOTICE ABOUT OPEN ACCESS: To assist authors whose funding agencies mandate immediate public access to published research findings, The Journal of Physiology allows authors to pay an Open Access (OA) fee to have their papers made freely available immediately on publication.

The Corresponding Author will receive an email from Wiley with details on how to register or log in to Wiley Authors where you will be able to place an order.

• You can check if your funder or institution has a Wiley Open Access Account here:
https://authors.wiley.com/author-resources/Journal-Authors/open-access/author-compliance-tool.html

MEMBER DISCOUNT: Corresponding authors who are Members of the Physiological Society are eligible for a 20% discount on the cost of the Article Processing Charge (APC). To claim this discount, the Corresponding Author will need to log in to the Physiological Society Membership portal and retrieve the discount code (https://www.physoc.org/journals-and-media/journals/journal-discount-for-members/). This code should then be quoted in the billing section of Wiley Authors when selecting the open access option.

• TRANSPARENT PEER REVIEW POLICY: To improve the transparency of its peer review process, The Journal of Physiology publishes online as supporting information the peer review history of all articles accepted for publication. Readers will have access to decision letters, including Editors' comments and referee reports, for each version of the manuscript as well as any author responses to peer review comments. Referees can decide whether or not they wish to be named on the peer review history document.

• You can help your research get the attention it deserves! Check out Wiley's free Promotion Guide for best-practice recommendations for promoting your work at: www.wileyauthors.com/eeo/guide. You can learn more about Wiley Editing Services which offers professional video, design, and writing services to create shareable video abstracts, infographics, conference posters, lay summaries, and research news stories for your research at: www.wileyauthors.com/eeo/promotion.

• If you would like to receive our 'Research Roundup', a monthly newsletter highlighting the cutting-edge research published in The Physiological Society's family of journals (The Journal of Physiology, Experimental Physiology, Physiological Reports, The Journal of Nutritional Physiology and The Journal of Precision Medicine: Health and Disease), please click this link, fill in your name and email address and select 'Research Roundup':
https://www.physoc.org/journals-and-media/membernews

EDITOR COMMENTS

Reviewing Editor:

Comments to the Author (Required):
I would like to accept this manuscript for publication.

Senior Editor:

Comments to the Author:
Thank you for the revision. Congratulations!
